# Improved prediction of brain age using multimodal neuroimaging data

**DOI:** 10.1002/hbm.24899

**Published:** 2019-12-14

**Authors:** Xin Niu, Fengqing Zhang, John Kounios, Hualou Liang

**Affiliations:** ^1^ Department of Psychology Drexel University Philadelphia Pennsylvania; ^2^ School of Biomedical Engineering, Science and Health Systems Drexel University Philadelphia Pennsylvania

**Keywords:** bias correction, brain age prediction, machine learning, multimodal brain imaging

## Abstract

Brain age prediction based on imaging data and machine learning (ML) methods has great potential to provide insights into the development of cognition and mental disorders. Though different ML models have been proposed, a systematic comparison of ML models in combination with imaging features derived from different modalities is still needed. In this study, we evaluate the prediction performance of 36 combinations of imaging features and ML models including deep learning. We utilize single and multimodal brain imaging data including MRI, DTI, and rs‐fMRI from a large data set with 839 subjects. Our study is a follow‐up to the initial work (Liang et al., 2019. *Human Brain Mapping*) to investigate different analytic strategies to combine data from MRI, DTI, and rs‐fMRI with the goal to improve brain age prediction accuracy. Additionally, the traditional approach to predicting the brain age gap has been shown to have a systematic bias. The potential nonlinear relationship between the brain age gap and chronological age has not been thoroughly tested. Here we propose a new method to correct the systematic bias of brain age gap by taking gender, chronological age, and their interactions into consideration. As the true brain age is unknown and may deviate from chronological age, we further examine whether various levels of behavioral performance across subjects predict their brain age estimated from neuroimaging data. This is an important step to quantify the practical implication of brain age prediction. Our findings are helpful to advance the practice of optimizing different analytic methodologies in brain age prediction.

## 1 INTRODUCTION

Brain maturity is a complex process that involves cortical thinning (Gogtay et al., 2004), synaptic pruning (Purves & Lichtman, 1980), and axon myelination (Benes, Turtle, Khan, & Farol, 1994) as a result of both genetics and postnatal experience. The developmental processes result in considerable and broadly structural changes of gray matter and white matter (Brown et al., 2012; Davis et al., 2009; Tamnes et al., 2010) as well as morphological (Corps & Rekik, 2019) and functional connectivity changes across the brain (Davis et al., 2009; Dosenbach et al., 2010). These changes are associated with various aspects of cognition, emotion, and mental disorders. For example, frontal lobe gray matter thinning has been found to be related to verbal and visuospatial memory (Sowell, Delis, Stiles, & Jernigan, 2001). Development of working memory capacity is positively correlated with frontal‐parietal connectivity (Nagy, Westerberg, & Klingberg, 2004). There is also evidence showing aberrant brain development in patients with psychiatric disorders such as autism (Hazlett et al., 2011; Hazlett et al., 2017; Schumann et al., 2010), dementia (Davatzikos, Xu, An, Fan, & Resnick, 2009) and schizophrenia (Gur et al., 1998; McGlashan & Hoffman, 2000). Thus, understanding brain development provides insights into the development of cognition and mental disorders.

### 1.1 The brain age gap

Brain age prediction has drawn great interest among researchers in recent years. Using machine learning (ML) methods, an age prediction model is first built with brain imaging features from a training data set and then applied to estimate the age of new individuals in an independent test set. It has been shown ML models can achieve a correlation coefficient between the predicted and chronological age of around .90 (Ashburner, 2007; Brown et al., 2012; Dosenbach et al., 2010; Franke, Ziegler, Klöppel, & Gaser, 2010). The predicted age based on brain imaging data is often referred to as brain age, which may serve as a potential biomarker for development‐related mental disorders or a brain development index for healthy individuals. The difference between predicted age and chronological age is often referred to as the brain age gap, which is helpful for quantifying delayed or advanced development for youth as well as accelerated or slowed aging for elderly people.

As an index of deviation from a healthy brain‐aging trajectory, the brain age gap has the potential to suggest age‐associated brain disease or cognitive aging with both neuroscientific and clinical implications (Cole & Franke, 2017). For example, accelerated brain aging was found in patients with Alzheimer's disease (Franke et al., 2010; Franke & Gaser, 2012), traumatic brain injury (Cole, Leech, & Sharp, 2015), and psychiatric disorders such as schizophrenia and major depression disorders (Chung et al., 2018; Koutsouleris et al., 2014). In a longitudinal study, accelerated brain aging was found to be an indicator of conversion from mild cognitive impairment to Alzheimer's disease (Gaser et al., 2013). For adults aged between 19 and 79 years, lower brain age suggests healthy aging that benefits from education and physical activity (Steffener et al., 2016), whereas for youth aged 8–21 years, higher brain age is related to better cognitive performance (Erus et al., 2015).

### 1.2 The bias of brain age estimates

Unfortunately, a systematic bias of the estimated brain age gap, manifested as a negative correlation between brain age gap and chronological age (Aycheh et al., 2018; Cole & Franke, 2017; Le et al., 2018; Pardoe & Kuzniecky, 2018), limits its potential applications. Specifically, brain age tended to be underestimated for older subjects and overestimated for younger ones, leading to systematic bias of the estimated brain age gap (Aycheh et al., 2018; Cole & Franke, 2017; Pardoe & Kuzniecky, 2018). The potential reasons for this bias are under debate, including regression toward the mean (Liang, Zhang, & Niu, 2019), and nongaussian distribution of subject ages (Smith, Vidaurre, Alfaro‐Almagro, Nichols, & Miller, 2019). This systematic bias tends to introduce a confounding effect of chronological age when the brain age gap is examined as a biomarker for a mental disease or cognition development. Recent studies used linear and nonlinear models to correct the bias in the brain age gap (Chung et al., 2018; Le et al., 2018; Liang et al., 2019).

The nonlinear relationship between the brain age gap and chronological age has not been explored thoroughly. Although a moderation effect of gender in brain development has been reported (Erus et al., 2015; Goyal et al., 2019), few studies took gender and its interaction with chronological age into consideration in bias correction of the brain age gap. Furthermore, most studies of age prediction used the brain age gap as a biomarker for brain aging or developmental delay. There was no direct comparison between brain age and chronological age in terms of their relationship with cognitive performance. This is an important step in brain age prediction to check if our proposed method is able to capture the cognitive behavior with brain age relative to chronological age.

### 1.3 Multimodal imaging features

Studies have investigated brain age prediction with features derived from single imaging modality, such as *T*_1_‐weighted MRI (Franke et al., 2010; Tamnes et al., 2010), diffusion tensor imaging (DTI) (Mwangi, Hasan, & Soares, 2013) and resting‐state fMRI (Dosenbach et al., 2010). It is promising that age prediction performance can be further improved with multimodal brain imaging features. There is evidence for the advantage of multimodal features in age prediction. For example, it has been shown that combining imaging features from all three modalities (*T*_1_‐ and *T*_2_‐weighted MRI as well as DTI) yielded higher prediction accuracy than did a single‐modality (Brown et al., 2012). The combination of *T*_1_‐weighted features and resting functional connectivity features also yielded higher prediction performance than single‐model features (Liem et al., 2017). The multimodal brain imaging data including *T*_1_‐weighted MRI, DTI, and resting‐state fMRI features has been used in brain age prediction (Liang et al., 2019). In this work, we follow up with the initial study (Liang et al., 2019) with more subjects.

### 1.4 Machine learning algorithms for estimating brain age

Different ML algorithms have been used for brain age prediction, which include support vector regression (SVR) (Dosenbach et al., 2010; Erus et al., 2015; Franke et al., 2010; Koutsouleris et al., 2014; Liem et al., 2017), Gaussian process regression (GPR) (Aycheh et al., 2018; Cole et al., 2015, 2017), relevance vector regression (RVR) (Franke & Gaser, 2012; Gaser et al., 2013; Mwangi et al., 2013), ridge regression (Chung et al., 2018) and elastic net (Khundrakpam, Tohka, & Evans, 2015). Overall, these ML models showed comparable performance on age prediction (Liang et al., 2019). It has been shown that SVR, RVR, ridge regression and elastic net performed similarly on the prediction of the motor and cognitive scores with resting‐state functional connectivity features (Cui & Gong, 2018). However, no studies systematically and directly compared them on age prediction. Deep neural networks (DNNs) have also been applied to brain age prediction recently. With recent advances in deep learning, DNNs are expected to improve prediction performance (Cole & Franke, 2017). However, most studies showed DNN yielded similar prediction performance to traditional ML methods (Aycheh et al., 2018; Cole et al., 2017). For example, a convolutional neural network (CNN) achieved comparable prediction accuracy (*r* = .96) to GPR (Cole et al., 2017). Though prediction performance was not significantly improved, one advantage is that DNN is directly applicable to raw imaging data. This makes DNN appropriate for brain images with gross pathology, for which standard imaging preprocessing pipeline does not perform well. In that study (Cole et al., 2017), only *T*_1_‐weighted MRI was used. It remains unknown whether DNN trained with multimodal imaging data outperforms traditional ML methods.

### 1.5 Linear and nonlinear estimations

So far, most studies only examined a linear relationship between chronological age and brain age and used Pearson's correlation to evaluate prediction performance (Erus et al., 2015; Franke et al., 2010; Goyal et al., 2019). It is well known that brain imaging features for adults do not change with chronological age as quickly as they do for adolescents. Studies have reported a nonlinear relationship between chronological age and multimodal brain imaging features of gray matter, white matter, and functional connectivity (Brown et al., 2012; Dosenbach et al., 2010; Schumann et al., 2010). To our knowledge, only one study examined a nonlinear relationship between chronological age and predicted brain age based on functional connectivity data (Dosenbach et al., 2010). No studies examined the potential nonlinear relationship between chronological age and predicted brain age using structural or multimodal brain imaging features. Therefore, in this study, we aim to investigate the nonlinear relationship between chronological age and brain age predicted by multimodal imaging features. As a linear relationship is not assumed, we propose to use the *R* ^2^ from quadratic regression between chronological age and brain age as a metric to evaluate model prediction performance. Furthermore, as true brain age is unknown and may deviate from chronological age, it is not recommended to use chronological age as the gold standard to evaluate model prediction accuracy. Therefore, we instead examine the proportion of explained variance in brain age by subjects' performance in various cognitive behavioral tasks.

### 1.6 Anxiety disorder

Anxiety disorder is known to be related to brain development in mice (Desrumaux et al., 2018; Fujita et al., 2017) and humans (Fuhrmann, Knoll, & Blakemore, 2015). Youth with post‐traumatic stress disorder (PTSD) showed increased amygdala activation with age and decreased connectivity between the amygdala and ventral medial prefrontal cortex in emotional tasks (Keding & Herringa, 2016; Wolf & Herringa, 2016). A recent longitudinal study revealed that youth with PTSD showed aberrant development of gray matter volume (GMV) in the dorsolateral prefrontal cortex (PFC), and decreased ventrolateral PFC‐hippocampus and ventromedial PFC‐amygdala connectivity (Heyn et al., 2018). In addition, patients with specific phobia showed higher activations in the medial prefrontal cortex, anterior cingulate cortex, amygdala, insula, and thalamus in response to phobia‐related conditioned stimuli (Del Casale et al., 2012). Response to social‐affective stimuli in the amygdala, striatum and the frontal areas of children with social anxiety disorder showed complex developmental trajectories in adolescents (Haller, Cohen Kadosh, Scerif, & Lau, 2015). However, little is known about whether these alterations reflect delayed development (Herringa, 2017). To further evaluate the performance of our proposed model for brain age prediction, we apply the optimized ML model to a group of adolescents with anxiety disorders.

### 1.7 Overview

In summary, we investigated brain age prediction with different ML models and brain features from multiple imaging modalities. Additionally, we considered a nonlinear relationship between chronological age and brain age and suggested a new approach to evaluate model performance by comparing the *R* ^2^ from a quadratic regression model. Furthermore, the quadratic regression model was employed to provide a way to correct for the bias of the brain age gap. We further examined whether the brain age and the bias‐corrected brain age gap are related to behavioral performance. Findings from our study have the potential to advance the practice of optimizing different analytic methodologies in brain age prediction. In addition, we applied the proposed brain age prediction model to a group of adolescents with anxiety disorders and tested whether patients with anxiety disorders showed altered brain development compared to healthy controls.

## 2 METHODS

### 2.1 Participants

Subjects were selected from the Philadelphia Neurodevelopmental Cohort (PNC) study (Gur et al., 2010). The PNC study is a large‐scale research initiative, aiming to understand how brain maturation and genetics mediate the development of cognition on healthy people and patients with psychiatric illness (Satterthwaite et al., 2014). Details of subject recruitment and study procedures can be found in published papers (Gur et al., 2010; Satterthwaite et al., 2014). After excluding 17 subjects with severe general medical problems, the current study selected 839 subjects (331 females) with multimodal brain imaging data available which include *T*_1_ weighted MRI, DTI, and resting‐state fMRI. These subjects were 8–21 years of age with a mean age of 14.20 years (*SD* = 3.29). Among them, there were 60 healthy controls (HCs; 29 females) aged 8–21 years (mean = 14.42, *SD* = 3.84), 70 subjects with PTSD (50 females) aged 9–21 years (mean = 15.51, *SD* = 2.74), 185 subjects with specific phobia (120 females) aged 8–21 years (mean = 13.60, *SD* = 3.19), and 142 subjects with social phobia (82 females) aged 8–21 years (mean = 14.46, *SD* = 2.83). The remaining subjects had other mental disorders such as depression, psychosis, and ADHD. The disorder symptoms were assessed using a computerized, structured interview (GOASSESS) that was administered to probands, caregivers or legal guardians depending on the age of subjects (Calkins et al., 2015). Data used in this study are publicly available through the database of Genotypes and Phenotypes (dbGaP). The R and Python codes for implementing ML methods in the paper are available upon request.

### 2.2 Computerized neurocognitive battery

All the participants completed a 1‐hr Penn Computerized Neurocognitive Battery (CNB), which consists of 14 neurocognitive tests including abstraction and mental flexibility (ABF), attention (ATT), working memory (WM), verbal memory (VMEM), face memory (FMEM), spatial memory (SMEM), language reasoning (LAN), nonverbal reasoning (NVR), spatial processing (SPA), emotion identification (EMI), emotion differentiation (EMD), age differentiation (AGD), sensorimotor processing speed (SM), and motor speed (MOT). All the tests include measures of both accuracy and speed except for the SM and MOT tests that only measure speed. In our analysis, the speed measures were multiplied by −1 to make higher value reflect better performance. A detailed description of tasks and assessment procedures can be found in a previous study (Gur et al., 2010).

### 2.3 MR image acquisition

Imaging data were acquired using a Siemens Tim Trio (Erlangen, Germany) 3T scanner equipped with 40 mT/m gradients and 200 mT/m/s slew‐rates. RF transmission utilized a quadrature body‐coil and reception using a 32‐channel head coil optimized for parallel imaging. The *T*_1_‐weighted protocol utilized a 3D, inversion‐recovery, magnetization‐ prepared rapid acquisition gradient echo (MPRAGE) with TI/TR/TE = 1100/1810/3.51 ms, flip angle = 9°, matrix = 256 × 192, FOV = 240 × 180 mm, slices = 160, and slice thickness = 1 mm. DTI images were acquired with a twice‐refocused spin‐echo single‐shot EPI sequence and a custom 64‐direction diffusion set, with *b*‐values of 0 and 1,000 s/mm^2^. The *b* = 0 scans were repeated 6 times, each *b* = 1,000 scan was acquired once at each direction, for a total of 70 repetitions. The acquisition parameters were TR/TE = 8,100/82 ms, matrix = 128 × 128, FOV = 240 mm, slices = 70, slice thickness = 2 mm and GRAPPA factor = 3. Resting‐state BOLD scans were acquired with a single‐shot, interleaved multi‐slice, gradient‐echo, echo planar imaging (GE‐EPI) sequence, with a repetition time (TR) = 3,000 ms. 46 slices with a voxel resolution of 3 × 3 × 3 mm were obtained. The total duration of the resting‐state fMRI was 6.3 min. More details of MRI scan protocols and scanner stability for *T*_1_‐weighted imaging, DTI and rs‐fMRI were described in a previous study (Satterthwaite et al., 2014).

### 2.4 Image preprocessing

The GMV was obtained from the *T*_1_‐weighted images with CAT v12.5 toolbox in SPM12 under MATLAB. The preprocessing steps included bias correction, affine registration, global intensity correction, and segmentation. Then Dartel registration was run with the MNI 152 template. The GMV was averaged based on the neuromophormetrics atlas. DTI images were preprocessed with a pipeline tool, PANDA (Cui, Zhong, Xu, He, & Gong, 2013). Processing steps included skull removal, correction of eddy current distortion, and building diffusion tensor models. Fractional anisotropy (FA) and mean diffusivity (MD) values were calculated and averaged based on the Johns Hopkins University (JHU) white‐matter tractography atlas. We chose the 25%‐threshold sub‐template which contains 20 major tracts and 50 major labels. Resting‐state fMRI data were processed using the software Data Processing Assistant for Resting‐State fMRI (DPARSF; Yan & Zang, 2010). Processing steps included slice timing correction, realignment, co‐registration with *T*_1_ image, segmentation, and normalization. Global signal regression was performed before extracting the signal. After obtaining the amplitude of low‐frequency fluctuation (ALFF) and regional homogeneity (ReHo), atlas‐based features were extracted based on the BN246 atlas (Fan et al., 2016).

### 2.5 The rationale of brain age prediction

Unlike other applications of ML studies, the outcome variable ‐true brain age ‐ was unknown. This naturally raises the question of why the predicted age is interpreted as brain age when chronological age was used to train the age prediction model. Here we clarified the rationale of brain age prediction using the following two equations. The first equation represents that true brain age (*y*_*b*_) differs from chronological age (*y*_*c*_) by a small amount (i.e., the brain age gap *ε*_1_).

$$y_{b}=y_{c}+\varepsilon_{1}$$

The second equation below illustrates that true brain age (*y*_*b*_) can be expressed as a function of different brain imaging features (*X*) with a small prediction error (*ε*_2_).

$$y_{b}=f\left(X\right)+\varepsilon_{2}$$

Combining the above two equations yields the following relationship between chronological age and the predicted brain age (*f*(*X*)).

$$y_{c}=f\left(X\right)-\varepsilon_{1}+\varepsilon_{2}$$

Thus, the estimated brain age gap (i.e., *f*(*X*) − *y*_*c*_) is a mixture of true brain age gap (*ε*_1_) and prediction error (*ε*_2_). When *ε*_1_ is relatively large and *ε*_2_ is relatively small, the estimated brain age gap is closer to true brain age gap. When *ε*_1_ is relatively small and *ε*_2_ is relatively large, the estimated brain age gap contains mostly prediction error. To confirm the estimated brain age gap is meaningful, we examined whether the brain age gap was significantly correlated with cognitive behavioral scores.

### 2.6 Machine learning methods

As previously done in Liang et al. (2019), we compared four popular ML models including ridge regression, SVR, GPR, and DNN with the chronological age as the outcome variable. Based on the above rationale, the predicted age was interpreted as the brain age. The scikit‐learn library v0.19 (Pedregosa et al., 2011) was used to implement ridge regression, SVR and GPR. DNN was implemented with PyTorch v0.4 (Paszke et al., 2017). Ridge regression minimizes the sum of squared prediction error and L_2_‐norm regularization term. Nested cross‐validation (CV), with 10‐fold outer CV and threefold inner CV, was conducted to evaluate the prediction performance. The regularization parameter *λ* controlling the bias‐variance trade‐off was tuned in the inner loop CV. In each outer CV, 90% of the data were selected as a training set, with the remaining as a test set. Feature normalization was conducted on the training set and then applied to the test set. As the robustness of prediction accuracy improves with a larger sample size (Cui & Gong, 2018), we used data from all 839 subjects for model evaluation and feature selection.

SVR (Smola & Schölkopf, 2004) aims to find a function based on a subset of the training samples (support vectors) by ignoring the prediction error which is less than a threshold (*ε*) while minimizing the complexity of the function. The current study used the radial basis function kernel which maps the original features into a higher dimensional space. This enables the model to fit a nonlinear relationship between imaging features and age. A parameter C, which controls the trade‐off between prediction accuracy on training samples and maximization of the decision function's margin, was tuned in the inner loop CV (Zhang, Wang, Kim, Todd, & Wong, 2015). Nested CV, parameter tuning, and feature normalization were conducted in a way similar to what has been described above for ridge regression.

GPR (Rasmussen, 2004) uses kernels to define the covariance of a prior distribution over the target functions and uses the observed training data to estimate a likelihood function. Based on Bayes theorem, a Gaussian posterior distribution over target functions is defined and its mean is used for prediction. We conducted the nested CV, parameter tuning, and feature normalization similar to what has been described above for ridge regression.

Deep learning refers to computational models composed of multiple layers of processing units (LeCun, Bengio, & Hinton, 2015). Each layer of neurons uses nonlinear modules to transform the representation of data to different levels of abstraction. This structure allows the neural network to learn hierarchical feature representations which makes it a useful tool to fit the complex brain growth patterns (Hazlett et al., 2017). DNNs with different structures were constructed for single and multimodal features. Hyperparameters (i.e., the number of neurons and regularization parameters in each layer) were tuned using CV. Data were randomly split into a training set (90% of the data) and a test set. This process was repeated 10 times with stratified shuffle split. Each time a different seed was used. The optimized loss values were averaged. After the hyperparameters were tuned, a 10‐fold CV was conducted to evaluate the model performance. The structure of DNN for GMV, ALFF, ReHo, and multimodal features had five hidden layers with 400, 150, 50, 30, and 5 units from the first to the last hidden layer, respectively. The first and last hidden layers were linear models with a rectified linear unit activation function. Other layers were linear models with a sigmoid activation function. In each CV, Nesterov stochastic gradient descent (Dozat, 2016) was used to train the model in multiple epochs until the loss was less than .5, with the initial learning rate of .001, weight decay of 1e‐3 and momentum of .9. To decrease random jittering of loss curve, a multistep learning rate was applied. The learning rate decayed by .5 when the epoch exceeded 500, 1,000, 1,500, 3,000 and 6,000 epochs. For FA and MD, the DNN had hidden layers with 100, 50, 20 units in each layer. Other parameters were tuned similarly as above.

To quantify the contribution of individual neuroimaging feature to age prediction, we extracted the absolute value of the coefficients of the ridge regression model using the multimodal brain imaging features. For SVR, GPR, and DNN, we removed one feature at a time from the model and calculated the change in prediction accuracy. A higher reduction of prediction accuracy indicated higher contribution to age prediction. Additionally, we examined the correlation of feature contribution across brain regions between each pair of the four ML models using Kendall's Tau.

### 2.7 Statistical analysis

To examine and compare different ML methods and brain imaging features from different modalities, we trained four ML models (ridge regression, SVR, GPR, DNN) with single‐modal and multimodal brain imaging features. The multimodal features included structural features (GMV, FA, and MD), a combination of GMV and rs‐fMRI features (GMV, ALFF, and ReHo), a combination of DTI and rs‐fMRI features (FA, MD, ALFF and ReHo), and a combination of all the single model features (GMV, FA, MD, ALFF, ReHo). This yielded 36 different combinations of ML models and imaging features, which were then systematically compared in this study. We used a large data set with 839 subjects to train and evaluate model performance. The large sample size was essential for prediction robustness (Cui & Gong, 2018). The prediction accuracy was computed using 10‐fold CV to avoid overfitting.

The optimal combination of the ML model and imaging features identified by the comparison outlined above was then used for brain age prediction for all 839 subjects. The predicted age was interpreted as brain age. The brain age gap was computed as the difference between brain age and chronological age. In accordance with previous studies, a positive brain age gap referred to advanced brain development. To account for the systematic bias in brain age prediction (Liang et al., 2019), the nonlinear brain development trajectory (Dosenbach et al., 2010), as well as potential gender difference (Goyal et al., 2019), we extended the linear model (Liang et al., 2019) by using the nonlinear formula below to control for the confounding effects of chronological age and gender. Importantly, to fit the formula below, brain age was estimated from an independent test set using brain imaging data and ML methods.

(1) $$\text{brain}\;\mathrm{age}=\beta_{0}+\beta_{1}*\mathrm{age}+\beta_{2}*{\mathrm{age}}^{2}+\beta_{3}*\text{gender}+\varepsilon$$

Where the term *age* was used to account for the systematic bias (i.e., brain age tended to be overestimated for younger subjects and underestimated for older ones). The quadratic term *age* ^2^ was included to model the nonlinear brain development trajectory. The potential gender difference in brain age was also modeled by adding the term *gender*. Thus, the term *ε* represented the difference between brain age and chronological age after controlling for the confounding factors including the linear and quadratic effects of chronological age and gender. We refer to it as the *corrected brain age gap*.

To further account for the gender difference in brain development trajectory, we proposed to add gender and its interactions with chronological age to the model below:

(2) $$\text{brain}\;\mathrm{age}=\beta_{0}+\beta_{1}*\mathrm{age}+\beta_{2}*{\mathrm{age}}^{2}+\beta_{3}*\text{gender}+\beta_{4}*\text{gender}*\mathrm{age}+\beta_{5}*\text{gender}*{\mathrm{age}}^{2}+\varepsilon$$

Where the interaction between *gender* and *age* denoted the gender difference in the linear brain development speeds. The interaction between *gender* and *age* ^2^ was used to account for the gender difference in the nonlinear brain development trajectory. The term *ε* was interpreted as the corrected brain age gap controlling for the effects of gender, linear and quadratic age terms as well as their interactions. The remaining terms were similar to what was outlined above for Formula (1).

Comparison of different brain age prediction models has been primarily based on metrics such as Pearson's correlation coefficient and mean absolute error (MAE) calculated between predicted brain age and chronological age in the literature (Erus et al., 2015; Franke et al., 2010; Goyal et al., 2019). As we incorporated the nonlinearity and gender difference in the bias correction step for brain age gap, we proposed to use *R* ^2^ of the fitting curve (i.e., formula (2)) for model comparison. In this case, the prediction evaluation and bias correction were unified in a single model. We then calculated the Pearson's correlation between the corrected or uncorrected brain age gap and chronological age to quantify the prediction bias. As gender difference is prominent in brain development (Erus et al., 2015; Goyal et al., 2019), the prediction performance was also separately evaluated for males and females to examine gender difference in brain development trajectory. It should be noted that brain age is likely to deviate from chronological age especially for disorder groups. Therefore, existing comparison metrics are limited by the fact there is no gold standard to evaluate brain age prediction performance as true brain age is unknown. As such, additional validation measures such as cognitive behavioral scores were used to evaluate the usefulness of the estimated brain age gap, as outlined below.

To examine whether brain age prediction reveals more information about cognitive development than chronological age, we conducted a stepwise linear regression to predict the brain age and chronological age using cognitive behavioral task scores. As the true brain age is unknown and may deviate from chronological age, this was an essential step to quantify the practical implication of brain age predicted by neuroimaging data. The *R* ^2^ of the stepwise regression was computed to examine whether the brain age was more closely related to behavioral performance than chronological age. In addition, we examined whether the corrected brain age gap (the *ε* term in Formula (2)) was associated with behavioral performance. The stepwise regression was conducted using the R package MASS (Venables & Ripley, 2002), with gender, the speed and accuracy scores of behavioral tasks in the CNB as predictors.

To illustrate the broad application of the analytic approach we developed, we applied the optimized brain age prediction model outlined above to a group of adolescents with anxiety disorders including specific phobia, social phobia, PTSD. To examine whether adolescents with anxiety disorders show different brain development trajectory compared to HCs, we compared their brain age gap predicted by our proposed model with that of HCs using two‐sample permutation test (Ludbrook & Dudley, 1998).

## 3 RESULTS

### 3.1 Multimodal features yielded the highest prediction performance

**Figure 1:**
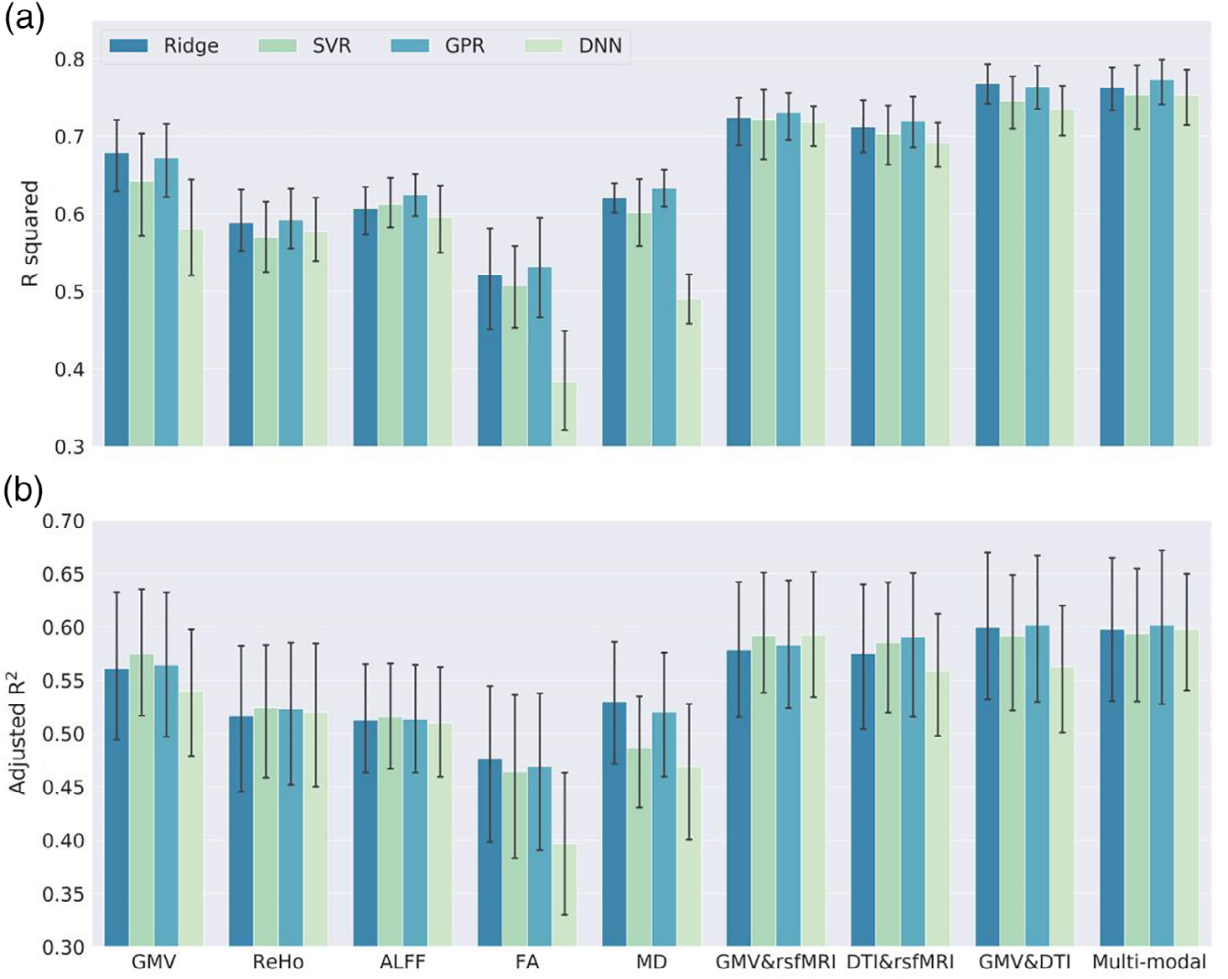
Prediction performance using all brain imaging modalities with four ML models and all subjects included. The multimodal brain imaging features yield higher prediction accuracy than any of the single modal features. (a) The prediction accuracy is defined as the R squared of quadratic regression with chronological age, gender and their interaction terms as predictors for the predicted age (brain age). The error bar is the 95% confidence interval generated by bootstrapping on the 10 folds cross‐validation. (b) The prediction performance is evaluated by the adjusted R squared of the stepwise regression using behavioral scores to predict brain age

We evaluated four popular ML models on single‐ and multimodal brain imaging features. The prediction performance on all samples (*N* = 839) of ridge regression, SVR, GPR, and DNN was summarized in Table 1. The result showed that multimodal brain imaging features achieved the best prediction performance across all four ML models (Figure 1a). The highest prediction accuracy obtained from 10‐fold CV was achieved by GPR using multimodal features (*R* ^2^ = .774, MAE = 1.384). The prediction accuracy of ridge regression (*R* ^2^ = .766, MAE = 1.414), SVR (*R* ^2^ = .756, MAE = 1.426), and DNN (*R* ^2^ = .753, MAE = 1.381) using multimodal brain imaging features were close to that of GPR (Figure 1a). In addition, we calculated the adjusted *R* ^2^ of stepwise regression models using behavioral scores to predict brain age (Figure 1b). Findings were consistent across the two panels of Figure 1. Among the unimodal features, GMV yielded the highest prediction performance, followed by MD, ALFF, and ReHo. The prediction performance with FA was the lowest among all the features. Additionally, though DNN yielded comparable prediction performance on multimodal features, it did not perform as well as traditional ML methods on unimodal features, especially for FA and MD that had a limited number of features.

**Table 1:** Prediction performance of four regression models on all samples

	Ridge				SVR				GPR				DNN			
	R squared		MAE		R squared		MAE		R squared		MAE		R squared		MAE	
GMV	0.678	(0.069)	1.662	(0.156)	0.640	(0.102)	1.699	(0.188)	0.673	(0.069)	1.649	(0.153)	0.581	(0.096)	1.837	(0.199)
ReHo	0.574	(0.075)	1.929	(0.207)	0.565	(0.069)	1.931	(0.197)	0.579	(0.073)	1.924	(0.211)	0.569	(0.068)	1.934	(0.163)
ALFF	0.606	(0.046)	1.788	(0.135)	0.608	(0.053)	1.803	(0.150)	0.621	(0.044)	1.770	(0.128)	0.592	(0.065)	1.811	(0.096)
FA	0.524	(0.103)	1.959	(0.171)	0.510	(0.082)	2.003	(0.164)	0.533	(0.097)	1.965	(0.159)	0.399	(0.107)	2.392	(0.145)
MD	0.608	(0.048)	1.829	(0.150)	0.587	(0.079)	1.949	(0.113)	0.621	(0.053)	1.845	(0.158)	0.474	(0.071)	2.177	(0.241)
GMV&DTI	0.772	(0.042)	1.396	(0.129)	0.747	(0.052)	1.440	(0.106)	0.768	(0.046)	1.395	(0.129)	0.737	(0.052)	1.436	(0.120)
GMV&rsfMRI	0.726	(0.049)	1.473	(0.132)	0.722	(0.070)	1.519	(0.123)	0.732	(0.051)	1.463	(0.143)	0.713	(0.045)	1.465	(0.129)
DTI&rsfMRI	0.715	(0.052)	1.550	(0.157)	0.706	(0.061)	1.520	(0.144)	0.722	(0.053)	1.512	(0.147)	0.694	(0.047)	1.520	(0.157)
Multimodal	0.766	(0.044)	1.414	(0.143)	0.756	(0.060)	1.426	(0.135)	0.774	(0.043)	1.384	(0.129)	0.753	(0.053)	1.381	(0.119)

*Note*: Prediction accuracy of age prediction is defined as the r squared of the quadratic fitting for prediction age (brain age) with chronological age and gender. Values in brackets are standard deviation across 10‐fold CV. MAE is the absolute value of the difference between the predicted age and chronological age.

**Figure 2:**
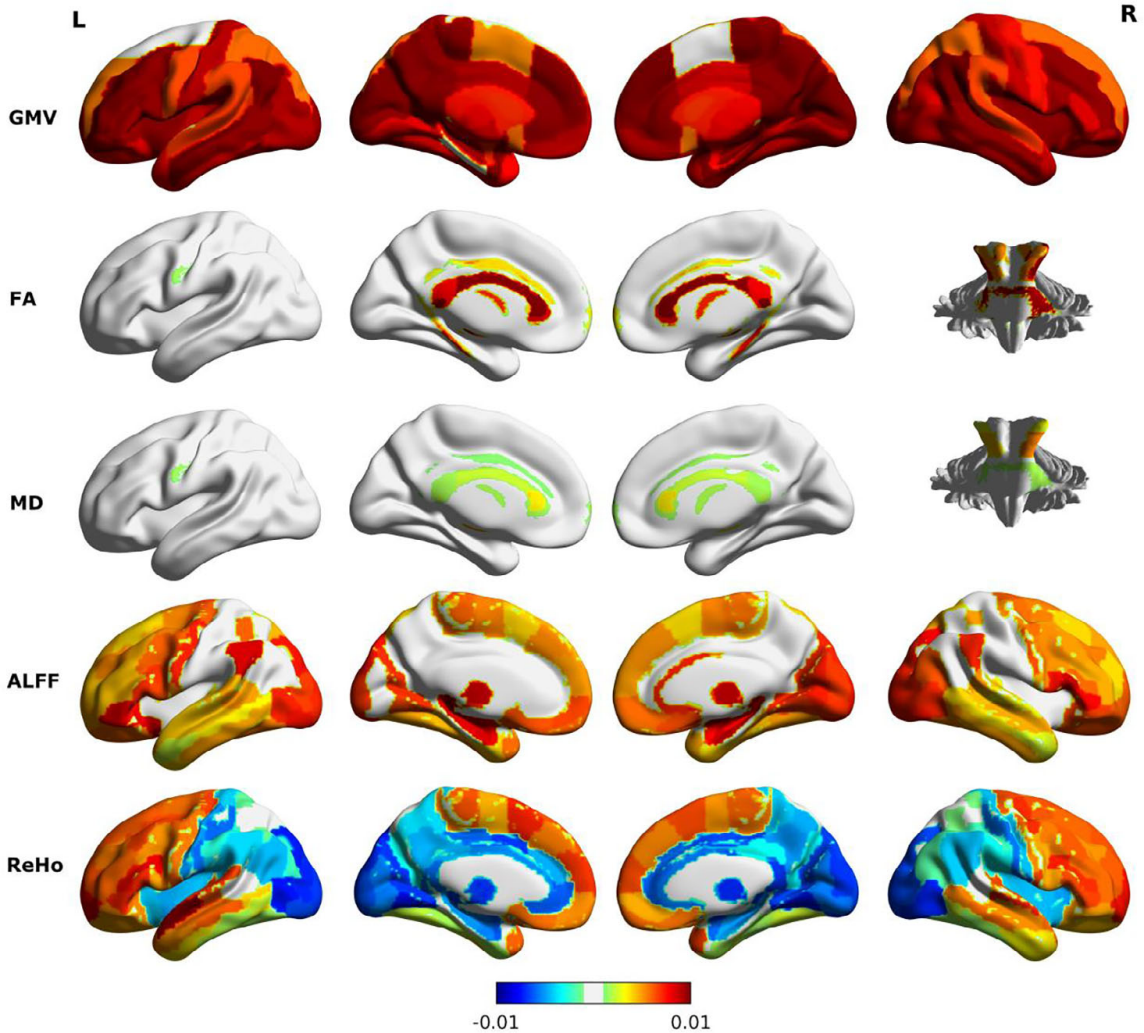
Brain imaging feature importance. The color bar indicates the feature importance that is the reduction of prediction accuracy (*R* ^2^ of the quadratic regression model) with each feature removed from the DNN model. Brain features with positive contribution to age prediction include GMV widely distributed across the whole brain, FA and MD in the corpus callosum and cerebellar peduncle, ALFF in the frontal and occipital cortex, and ReHo in the frontal and temporal cortex. ReHo features in the parietal and occipital region contribute negatively to age prediction

Brain imaging feature importance obtained from the DNN model was visualized in Figure 2. GMV widely across the brain contributed positively to brain age prediction. This suggests that removing individual GMV feature from the DNN model led to a large reduction of prediction accuracy. Features in other modalities with a positive contribution to brain age prediction were also widely distributed across the brain. ReHo in the occipital and parietal cortex had a negative contribution to brain age prediction. Comparison of neuroimaging feature importance identified across different ML methods showed consistent patterns across Ridge, SVR, and GPR (Figure S1). The feature contribution of DNN had a relatively low correlation with those obtained from other ML models. The predictive imaging features that were commonly selected across the four ML methods include the following: (a) GMV of the bilateral pallidum, precuneus, hippocampus, right posterior cingulate gyrus, posterior insula, and temporal pole, (b) FA of the corpus callosum, corticospinal tract, external capsule, longitudinal fasciculus, internal capsule, cerebellar peduncle, and posterior thalamic radiation, (c) MD of the bilateral cerebral peduncle, left posterior internal capsule, posterior corona radiata, and cingulum, (d) ALFF of the bilateral inferior frontal gyrus, left amygdala, cingulate gyrus, right insular gyrus, fusiform gyrus, inferior parietal lobe, and superior frontal gyrus, and (e) ReHo of the right superior temporal gyrus, superior frontal gyrus, left precentral gyrus, hippocampal gyrus, postcentral gyrus, and middle frontal gyrus. These were commonly ranked as top imaging features that contributed positively to brain age prediction. Top 100 neuroimaging features contributed positively to brain age prediction across the four ML models were summarized in Tables S1–S5.

### 3.2 The brain age gap with bias correction was orthogonal to chronological age

**Figure 3:**
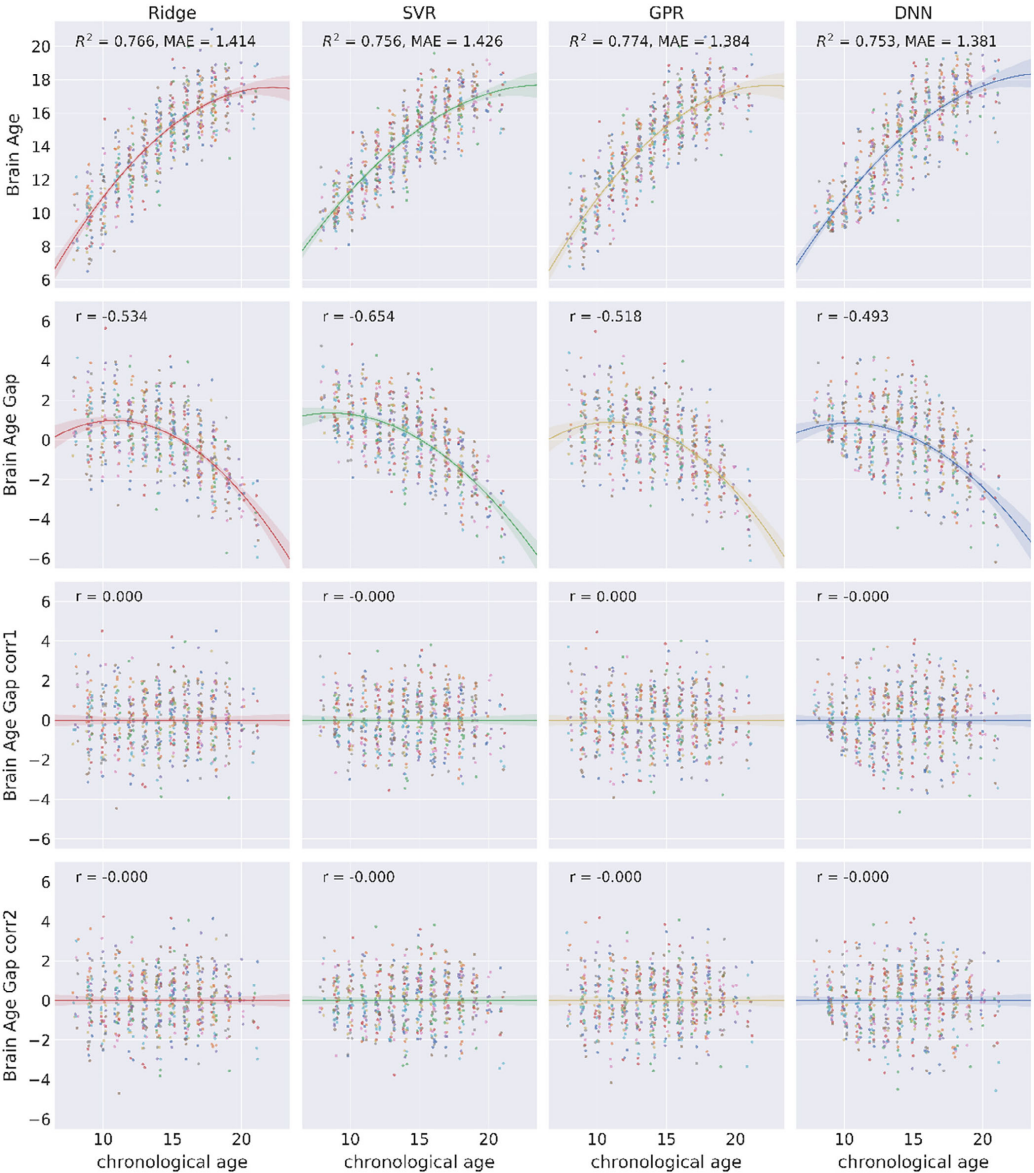
Scatterplots of brain age/brain age gap (*y*‐axis) and chronological age (*x*‐axis) of four ML models. The first row shows the relationship between the brain age and chronological age on multimodal brain imaging features. Color indicates samples in different folds of cross‐validation. The second row shows a negative correlation between the brain age gap (predicted age–chronological age) and chronological age. The third and fourth row show the corrected brain age gap is orthogonal to chronological age. The corrected brain age gap is the difference between the brain age and the fitted brain age with a quadratic function of chronological age, gender, and with (fourth row) or without (third row) their interaction terms

**Figure 4:**
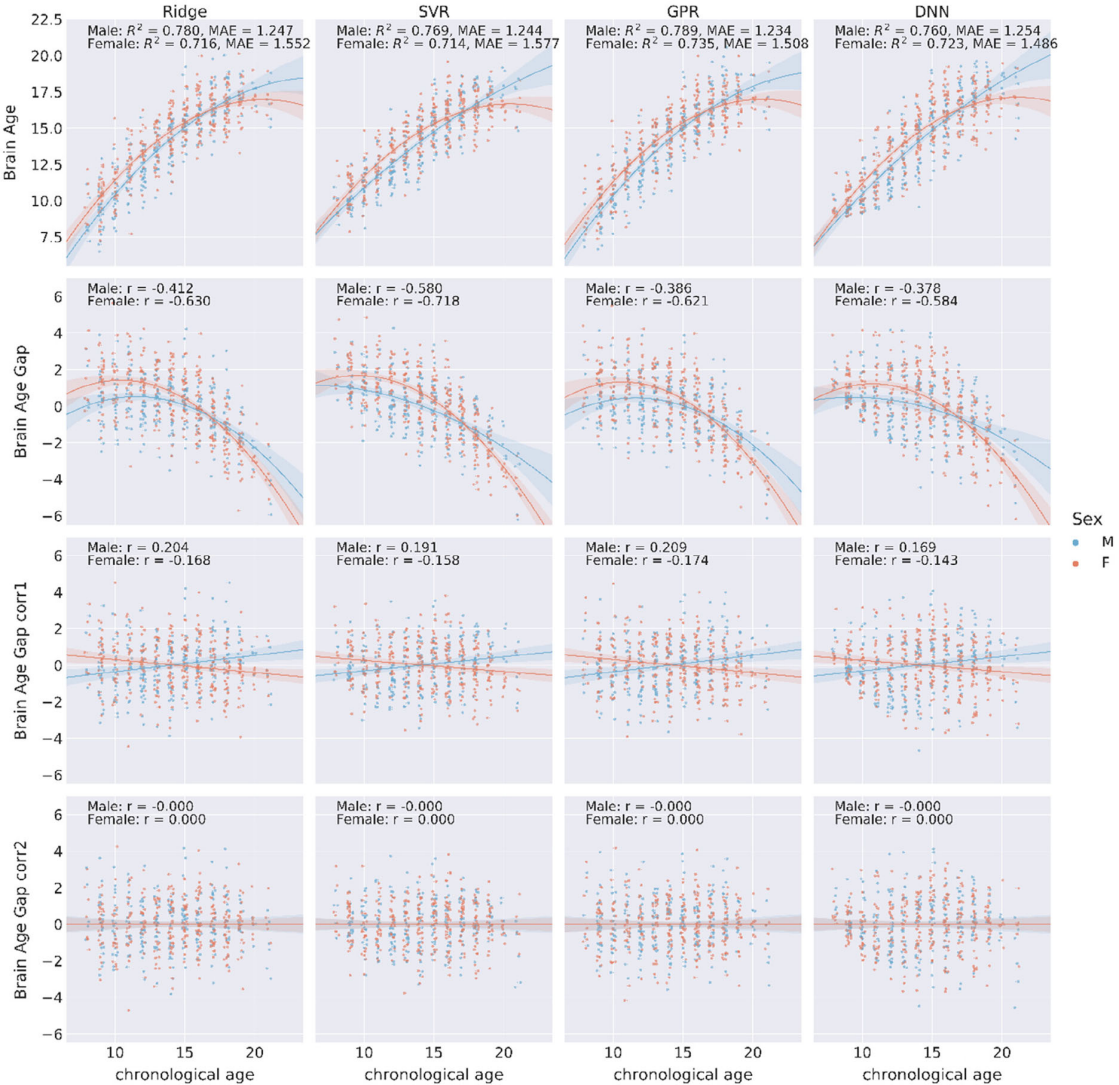
Scatterplots of brain age/brain age gap (*y*‐axis) and chronological age (*x*‐axis) of four ML models separately for males and females. The first row shows the brain age and chronological age for males and females. The second row shows a negative correlation between the brain age gap and chronological age. The third row shows the brain age gap with bias correction model without interaction terms. The fourth row shows that with interaction terms of gender and chronological age in the bias correction model, the corrected brain age gap is orthogonal to chronological age for both males and females

As shown in Figure 3, there was a clear nonlinear relationship between brain age gap and chronological age. Thus, we used a quadratic model to correct for the systematic bias of the brain age gap with gender and chronological age (i.e., Formula (1)). Residuals from the fitted model were interpreted as the corrected brain age gap. The corrected brain age gap was orthogonal to chronological age (Figure 3). However, when we examined males and females separately, the bias still existed. The corrected brain age gap was positively correlated with the chronological age for males and negatively correlated to the chronological age for females (Figure 4). To further remove this bias embedded in gender, we added interaction terms of gender and chronological age in the quadratic model (i.e., Formula (2)). With interaction terms in the bias correction, the brain age gap was orthogonal to chronological age for both males and females (fourth row in Figure 4).

### 3.3 Prediction of brain age with the behavioral performance for all subjects

After the brain age was obtained, we examined how the brain age and chronological age were associated with behavioral performance. Stepwise linear regression was conducted with the gender and CNB scores to predict brain age and chronological age separately. We used the adjusted *R* ^2^ to examine the predictability of CNB scores for brain age and chronological age. As shown in Table 2, the adjusted *R* ^2^ of the stepwise regression for chronological age was larger than that for the brain age predicted by the four ML models. The accuracy scores of attention (ATT, *p* < .01) and age differentiation (ADI, *p* < .001) tasks as well as the speed scores of attention (ATT, *p* < .001) and spatial memory (SMEM, *p* < .05) tasks were significant positive predictors for brain age. The speed score of sensory‐motor (SM, *p* < .001) task was a significant negative predictor for brain age. This result was consistent across all four ML models. There were more significant predictors for chronological age than those for brain age. The accuracy scores of verbal memory (VMEM, *b* = −.087, *p* < .05) and emotion identification (EDI, *b* = .191, *p* < .05) as well as the speed scores of face memory (FMEM, *b* = −.201, *p* < .05) and nonverbal reasoning (NVR, *b* = −.110, *p* < .05) tasks were significant predictors for chronological age. However, none of them was selected in the stepwise regression model for brain age. The ADI speed score was a significant negative predictor for brain age estimated from SVR and DNN, but not for chronological age.

**Table 2:** Stepwise linear regression on brain age and chronological age for all subjects

	Ridge		SVR		GPR		DNN		Chronological age	
	Estimate	Pr(>|*t*|)	Estimate	Pr(>|*t*|)	Estimate	Pr(>|*t*|)	Estimate	Pr(>|*t*|)	Estimate	Pr(>|*t*|)
(intercept)	0.271	**0.006**	0.244	**0.012**	0.240	**0.020**	0.128	0.287	0.012	0.915
SexM	−0.058	**0.000**	−0.072	**0.000**	−0.059	**0.000**	−0.077	**0.000**	−0.055	**0.000**
Accuracy_ABF	−0.054	0.067	−0.063	0.056	−0.060	0.054	−0.078	0.054	−0.067	**0.050**
Accuracy_ADI	0.317	**0.000**	0.357	**0.000**	0.341	**0.000**	0.468	**0.000**	0.383	**0.000**
Accuracy_ATT	0.129	**0.002**	0.153	**0.001**	0.146	**0.001**	0.178	**0.002**	0.157	**0.001**
Accuracy_EDI	‐	‐	‐	‐	‐	‐	‐	‐	0.191	**0.018**
Accuracy_LAN	0.057	0.133	0.094	**0.015**	0.068	0.087	0.102	**0.034**	0.129	**0.003**
Accuracy_SPA	0.063	0.080	‐	‐	0.066	0.079	‐	‐	0.068	0.104
Accuracy_VMEM	‐	‐	‐	‐	‐	‐	‐	‐	−0.087	**0.043**
Speed_ABF	−0.091	0.148	‐	‐	−0.093	0.158	‐	‐	‐	‐
Speed_ADI	−0.106	0.109	−0.162	**0.022**	−0.122	0.077	−0.181	**0.039**	‐	‐
Speed_ATT	0.311	**0.000**	0.366	**0.000**	0.335	**0.000**	0.471	**0.000**	0.418	**0.000**
Speed_FMEM	‐	‐	‐	‐	‐	‐	‐	‐	−0.201	**0.041**
Speed_MOT	−0.077	0.122	‐	‐	−0.075	0.151	‐	‐	−0.085	0.149
Speed_NVR	‐	‐	‐	‐	‐	‐	‐	‐	−0.110	**0.020**
Speed_SM	−0.364	**0.000**	−0.416	**0.000**	−0.374	**0.000**	−0.525	**0.000**	−0.424	**0.000**
Speed_SMEM	0.120	**0.014**	0.163	**0.001**	0.138	**0.007**	0.170	**0.008**	0.112	0.066
Speed_VMEM	0.142	0.099	‐	‐	0.143	0.114	‐	‐	0.324	**0.005**
Adjusted *R* ^2^	0.488		0.481		0.499		0.483		0.603	

*Note*: Values in the table are regression coefficients of stepwise linear regression with the brain age as the dependent variable. The last row is the adjusted R squared of the regression model. Empty values indicate the predictors are not selected in the model. Bold values indicate statistically significant difference (*p*‐value ≤0.01 ‐ ≥0.001) and (p‐value ≤0.001).

We then applied stepwise linear regression to predict brain age gap (with bias correction using Formula (2)) with gender and CNB scores. A significant association between CNB scores and corrected brain age gap controlling for gender would suggest that the corrected brain age gap contains meaningful information on brain development. The corrected brain age gap of SVR shared the largest variance with behavioral scores (Table 3). The accuracy score of language (LAN, *p* < .001), the speed scores of attention (ATT, *p* < .001), and verbal memory (VMEM, *p* < .01) tasks were significant negative predictors for brain age gap. The speed scores of face memory (FMEM, *p* < .001) and sensory‐motor (SM, *p* < .001) tasks were significant positive predictors for brain age. These results were consistent across all four ML methods. As the brain age gap estimated from SVR shared the largest variance with behavioral scores, we used brain age calculated from SVR for the following analyses.

**Table 3:** Stepwise linear regression on the bias‐corrected brain age gap for all subjects

	Ridge		SVR		GPR		DNN	
	Estimate	Pr(>|*t*|)	Estimate	Pr(>|*t*|)	Estimate	Pr(>|*t*|)	Estimate	Pr(>|*t*|)
(intercept)	0.254	**0.002**	0.361	**0.000**	0.242	**0.006**	0.525	**0.000**
SexM	0.046	**0.000**	0.051	**0.000**	0.046	**0.000**	0.047	**0.000**
Brain age	0.715	**0.000**	0.715	**0.000**	0.698	**0.000**	0.573	**0.000**
Accuracy_ADI	‐	‐	−0.210	**0.000**	‐	‐	−0.201	**0.000**
Accuracy_ATT	‐	‐	−0.065	0.088	‐	‐	−0.074	0.053
Accuracy_EDI	−0.160	**0.000**	‐	‐	−0.162	**0.000**	‐	‐
Accuracy_LAN	−0.141	**0.000**	−0.126	**0.000**	−0.140	**0.000**	−0.136	**0.000**
Accuracy_VMEM	0.059	0.063	0.059	0.075	0.061	0.068	0.064	0.053
Speed_ATT	−0.217	**0.000**	−0.207	**0.000**	−0.225	**0.000**	−0.197	**0.000**
Speed_EDI	‐	‐	‐	‐	‐	‐	0.062	0.098
Speed_FMEM	0.191	**0.009**	0.253	**0.001**	0.195	**0.009**	0.177	**0.018**
Speed_NVR	0.052	0.134	‐	‐	‐	‐	‐	‐
Speed_SM	0.219	**0.000**	0.233	**0.000**	0.251	**0.000**	0.233	**0.000**
Speed_SMEM	−0.063	0.156	‐	‐	‐	‐	‐	‐
Speed_VMEM	−0.241	**0.004**	−0.323	**0.000**	−0.283	**0.001**	−0.331	**0.000**
Speed_WM	0.095	0.057	‐	‐	0.100	0.061	‐	‐
Adjusted *R* ^2^	0.423		0.449		0.402		0.444	

*Note*: Values in the table are regression coefficients of stepwise linear regression with the brain age gap as the dependent variable. The last row is the adjusted R squared of the regression model. Empty values indicate the predictors are not selected in the model. Bold values indicate statistically significant difference (*p*‐value ≤0.01 ‐ ≥0.001) and (p‐value ≤0.001).

### 3.4 Brain age prediction for HCs and anxiety disorder patients

**Figure 5:**
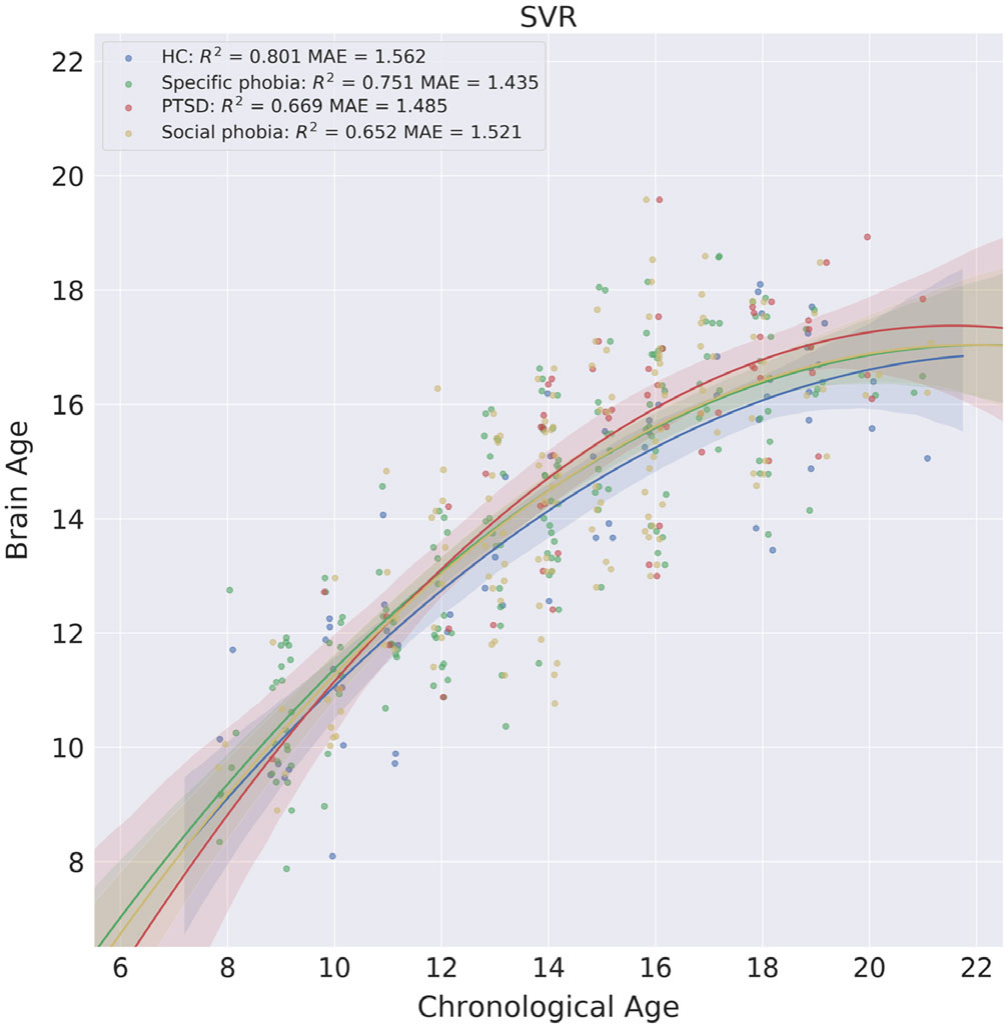
Scatterplots of the brain age and chronological age for the HC and anxiety disorder groups. The shaded area along the regression curve indicates the 95% bootstrapped confidence interval

In addition, we compared prediction performance between HCs and each of the three anxiety disorder groups. Similarly, we observed a nonlinear trend between chronological age and brain age (Figure 5). We calculated the prediction accuracy for HCs (*R* ^2^ = .801, MAE = 1.562), the specific phobia group (*R* ^2^ = .751, MAE = 1.435), the PTSD group (*R* ^2^ = .669, MAE = 1.485), and the social phobia group (*R* ^2^ = .652, MAE = 1.521).

To examine how brain age was differently related to behavioral performance for HCs and different disorder groups, we conducted a stepwise regression with gender and behavioral scores to predict brain age for each group separately. For HCs, the accuracy score of emotion differentiation (EDI, *b* = .716, *p* = .010) task, the speed scores of attention (ATT, *b* = .270, *p* = .043) and emotion differentiation (EDI, *b* = .371, *p* = .030) tasks were significant positive predictors (Table 4). There were no significant negative predictors for HCs. For specific phobia, the accuracy scores of age differentiation (ADI, *b* = .449, *p* = .002) and emotion identification (EID, *b* = .151, *p* = .028) tasks, and the speed score of attention (ATT, *b* = .475, *p* < .001) task were significant positive predictors. The accuracy scores of verbal memory (VMEM, *b* = −.157, *p* = .043) and sensory motor (SM, *b* = −.263, *p* = .005) tasks and gender (*b* = −.103, *p* < .001) were significant negative predictors. For PTSD, the accuracy scores of attention (ATT, *b* = .427, *p* = .013), face memory (FMEM, *b* = .317, *p* = .010), and working memory (WM, *b* = .312, *p* = .016) tasks, and the speed scores of attention (ATT, *b* = .365, *p* < .012) and motor praxis (MOT, *b* = .452, *p* = .009) tasks were significant positive predictors. The accuracy scores of abstract flexibility (ABF, *b* = −.312, *p* = .016), nonverbal reasoning (NVR, *b* = −.278, *p* = .027), spatial ability (SPA, *b* = −.239, *p* = .027), and verbal memory (VMEM, *b* = −.275, *p* = .015) tasks, and the speed score of age differentiation (ADI, *b* = −.232, *p* = .015) task were significant negative predictors. For social phobia, the accuracy score of emotion identification (EID, *b* = .264, *p* = .017) and the speed score of attention (ATT, *b* = .357, *p* = .005) task were significant positive predictors. The speed score of sensory motor (SM, *b* = −.373, *p* = .002) task and gender (*b* = −.116, *p* < .001) were significant negative predictors.

**Table 4:** Stepwise linear regression on the brain age for healthy control and disorder groups (SVR)

	HC		Specific phobia		PTSD		Social phobia	
	Estimate	Pr(>|*t*|)	Estimate	Pr(>|*t*|)	Estimate	Pr(>|*t*|)	Estimate	Pr(>|*t*|)
(intercept)	0.387	0.102	0.110	0.516	0.297	0.327	0.301	0.069
SexM	−0.115	0.054	−0.103	**0.000**	‐	‐	−0.116	**0.000**
Accuracy_ABF	‐	‐	‐	‐	−0.228	**0.046**	‐	‐
Accuracy_ADI	−0.433	0.126	0.449	**0.002**	‐	‐	‐	‐
Accuracy_ATT	‐	‐	‐	‐	0.427	**0.013**	‐	‐
Accuracy_EDI	0.716	**0.010**	−0.262	0.071	‐	‐	0.264	**0.017**
Accuracy_EID	0.186	0.135	0.151	**0.028**	0.169	0.152	‐	‐
Accuracy_FMEM	‐	‐	‐	‐	0.317	**0.010**	0.137	0.100
Accuracy_NVR	‐	‐	0.124	0.094	−0.278	**0.027**	‐	‐
Accuracy_SPA	‐	‐	‐	‐	−0.239	**0.027**	‐	‐
Accuracy_VMEM	‐	‐	−0.157	**0.043**	−0.275	**0.015**	‐	‐
Accuracy_WM	‐	‐	−0.123	0.127	0.312	**0.016**	‐	‐
Speed_ABF	‐	‐	‐	‐	‐	‐	−0.164	0.067
Speed_ADI	−0.219	0.152	‐	‐	−0.323	**0.015**	‐	‐
Speed_ATT	0.270	**0.043**	0.475	**0.000**	0.365	**0.012**	0.357	**0.005**
Speed_EDI	0.371	**0.030**	−0.139	0.097	0.225	0.059	‐	‐
Speed_LAN	‐	‐	0.235	0.071	‐	‐	‐	‐
Speed_MOT	‐	‐	−0.153	0.077	0.452	**0.009**	‐	‐
Speed_SM	−0.334	0.102	−0.263	**0.005**	−0.308	0.062	−0.373	**0.002**
Speed_SMEM	‐	‐	‐	‐	‐	‐	0.152	0.118
Speed_SPA	‐	‐	‐	‐	−0.217	0.080	‐	‐
Speed_VMEM	‐	‐	0.228	0.069	‐	‐	‐	‐
Speed_WM	‐	‐	0.181	0.089	−0.268	0.171	‐	‐
Adjusted *R* ^2^	0.491		0.539		0.514		0.417	

*Note*: Bold values indicate statistically significant difference (*p*‐value ≤0.01 ‐ ≥0.001) and (p‐value ≤0.001).

We further examined how brain age gap with bias correction using Formula (2) was related to behavioral performance controlling for brain age. We conducted a stepwise regression with brain age, gender and behavioral scores to predict the bias‐corrected brain age gap for each group separately. The result showed that the model explained largest variance of the corrected brain age gap for HCs (adjusted *R* ^2^ = .904). As shown in Table 5, for HCs, the accuracy scores of emotion differentiation (EDI, *b* = .242, *p* < .001) and language (LAN, *b* = .148, *p* = .011) tasks, and the speed scores of attention (ATT, *b* = .212, *p* < .004) and verbal memory (VMEM, *b* = .129, *p* = .046) tasks were significant positive predictors. The speed score of abstract flexibility (ABF, *b* = −.115, *p* < .037) was a significant negative predictor. For specific phobia, the accuracy score language (LAN, *b* = .154, *p* = .002) task and speed scores of the verbal memory (VMEM, *b* = .252, *p* = .001) task were significant positive predictors. The accuracy score of verbal memory (VMEM, *b* = −.150, *p* = .003) task and speed score of sensory motor (SM, *b* = −.130, *p* < .025) task were significant negative predictors. For PTSD, the accuracy scores of age differentiation (ADI, *b* = .200, *p* < .001) and nonverbal reasoning (NVR, *b* = .204, *p* = .005) tasks as well as the speed scores of age differentiation (ADI, *b* = .198, *p* < .006), attention (ATT, *b* = .302, *p* = .001) and verbal memory (VMEM, *b* = −.191, *p* < .049) tasks were significant positive predictors. The accuracy score of face memory (FMEM, *b* = −.127, *p* = .042) task and speed score of emotion differentiation (EDI, *b* = −.185, *p* < .018) task were significant negative predictors. For social phobia, the accuracy scores of age differentiation (ADI, *b* = .210, *p* = .004), attention (ATT, *b* = .158, *p* = .028), emotion identification (EID, *b* = .131, *p* = .020) tasks and the speed score of attention (ATT, *b* = .283, *p* = .003), and verbal memory (VMEM, *b* = .196, *p* = .050) tasks were significant negative predictors. The accuracy score of verbal memory (VMEM, *b* = −.130, *p* = .012) as well as the speed scores of face memory (FMEM, *b* = −.171, *p* = .038) and language (LAN, *b* = −.208, *p* = .020) tasks were significant negative predictors.

**Table 5:** Stepwise regression on the bias‐corrected brain age gap for HC and disorder groups (SVR)

	HC		Specific phobia		PTSD		Social phobia	
	Estimate	Pr(>|*t*|)	Estimate	Pr(>|*t*|)	Estimate	Pr(>|*t*|)	Estimate	Pr(>|*t*|)
(intercept)	−0.149	**0.017**	0.072	0.424	−0.285	**0.004**	0.092	0.503
SexM	‐	‐	−0.068	**0.000**	−0.050	0.079	−0.046	**0.049**
Brain age	0.606	**0.000**	0.654	**0.000**	0.620	**0.000**	0.546	**0.000**
Accuracy_ABF	‐	‐	‐	‐	0.098	0.095	‐	‐
Accuracy_ADI	‐	‐	‐	‐	0.200	**0.001**	0.210	**0.004**
Accuracy_ATT	‐	‐	0.071	0.150	‐	‐	0.158	**0.028**
Accuracy_EDI	0.242	**0.000**	‐	‐	‐	‐	‐	‐
Accuracy_EID	‐	‐	‐	‐	‐	‐	0.131	**0.020**
Accuracy_FMEM	‐	‐	‐	‐	−0.127	**0.042**	0.106	0.071
Accuracy_LAN	0.148	**0.011**	0.154	**0.002**	‐	‐	‐	‐
Accuracy_NVR	‐	‐	‐	‐	0.204	**0.005**	‐	‐
Accuracy_SPA	0.090	0.082	‐	‐	0.087	0.130	0.076	0.121
Accuracy_VMEM	‐	‐	−0.150	**0.003**	‐	‐	−0.130	**0.012**
Speed_ABF	−0.115	**0.037**	‐	‐	−0.102	0.127	‐	‐
Speed_ADI	‐	‐	−0.093	0.077	0.198	**0.006**	‐	‐
Speed_ATT	0.212	**0.004**	0.118	0.099	0.302	**0.001**	0.283	**0.003**
Speed_EDI	‐	‐	‐	‐	−0.185	**0.018**	‐	‐
Speed_FMEM	‐	‐	−0.122	0.072	−0.136	0.084	−0.171	**0.038**
Speed_LAN	‐	‐	‐	‐	‐	‐	−0.208	**0.020**
Speed_NVR	‐	‐	‐	‐	0.142	0.052	‐	‐
Speed_SM	‐	‐	−0.130	**0.025**	‐	‐	−0.160	0.070
Speed_VMEM	0.129	**0.046**	0.252	**0.001**	0.191	**0.049**	0.196	**0.050**
Speed_WM	−0.092	0.074	‐	‐	‐	‐	‐	‐
Adjusted *R* ^2^	0.904		0.811		0.834		0.757	

**Figure 6:**
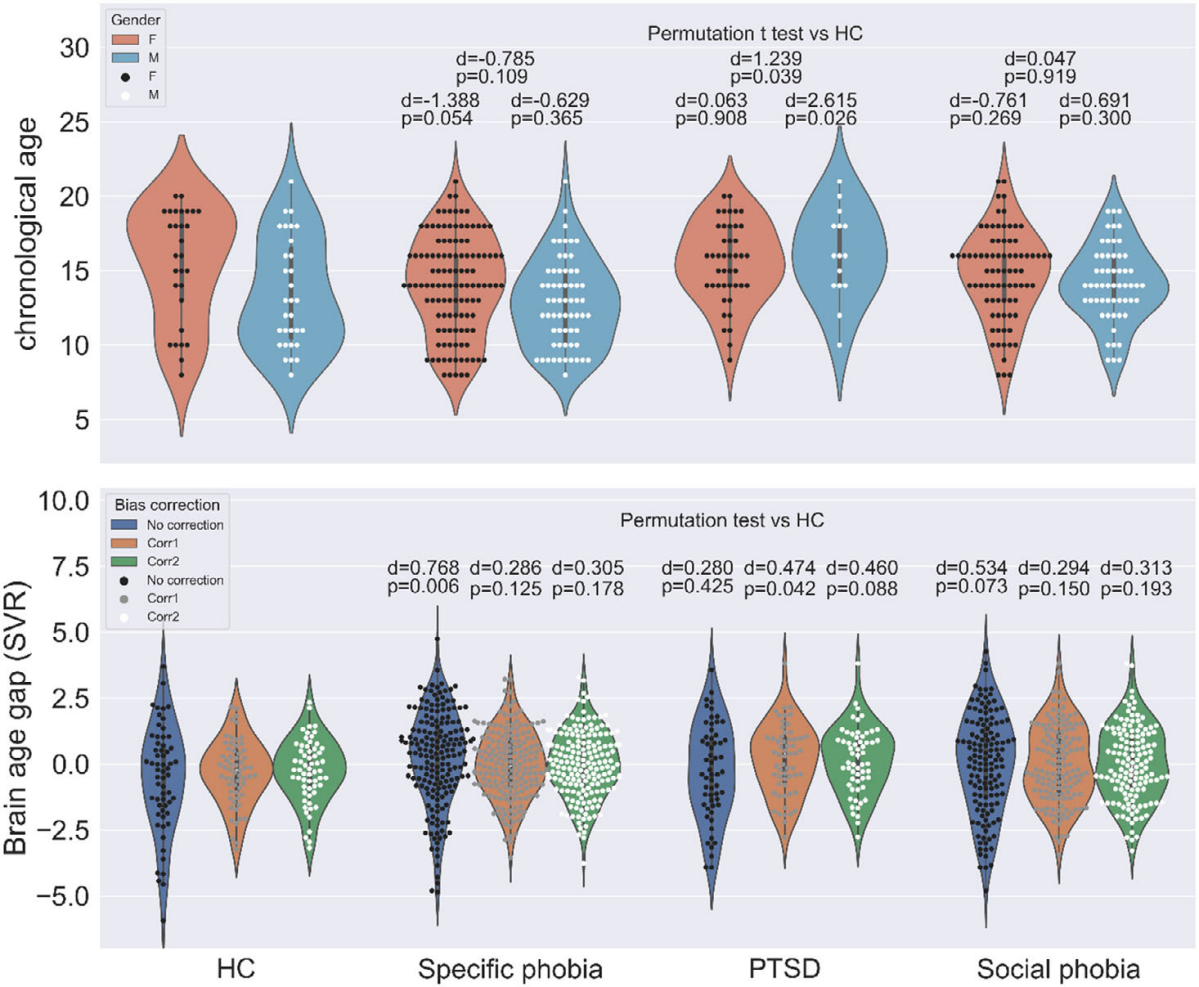
Chronological age and brain age gap for HC and groups with anxiety disorders. Chronological age of the PTSD group is significantly higher than that for the HCs for males (*p* < .05). Without bias correction, the brain age gap is significantly higher than HCs for the specific phobia group (*p* < .01) and marginally significantly higher than HCs for the social phobia group (*p* = .073). Using bias correction without interaction terms, the brain age gap for the PTSD group is significantly higher than HCs (*p* = .042). When interaction terms of gender and chronological age is included in bias correction, the result is marginally significant (*p* = .088)

To compare each disorder group with HCs, we conducted a permutation test on chronological age, uncorrected brain age gap, corrected brain age gap using Formulas (1) and (2). As shown in Figure 6, for the specific phobia group, the chronological age of females was marginally significantly lower than that in HCs (*d* = −1.388, *p* = .054). The uncorrected brain age gap was significantly higher than that of HC (*d* = .768, *p* < .01). With bias correction, the brain age gap was not significantly different from that of the HCs, regardless of which formula was used. For the PTSD group, the chronological age of males in the PTSD group was significantly higher than that in HCs (*d* = 2.615, *p* = .026). The uncorrected brain age gap was not significantly different from that of HCs (*d* = .280, *p* = .425). The corrected brain age gap using Formula (1) was significantly higher than that of HCs (*d* = .474, *p* = .042). The corrected brain age gap using Formula (2) was marginally significantly higher than that of HCs (*d* = .460, *p* = .088). For the social phobia group, no comparisons were significant.

To further evaluate our bias correction approach for brain age prediction, we conducted additional analyses with two separate parts. In the first part, we selected the HCs in the PNC study (*n* = 60) and built all four ML models with multimodal brain imaging features. The ML models were trained and tested using threefold CV. Findings were similar to what was described above. As shown in Figure S2, there was a nonlinear relationship between brain age and chronological age. After applying our bias correction methods, the corrected brain age gap was orthogonal to chronological age. In the second part of the analyses, we applied the ML models trained using only HCs to independent test sets (i.e., data from three disorder groups including specific phobia, PTSD, and social phobia). Figure S3 showed a nonlinear relationship between brain age and chronological age across different ML models and disorder groups. The consistent findings on the systematic bias of the estimated brain age gap suggested the importance of bias correction for brain age prediction.

## 4 DISCUSSION

In this work, we showed that multimodal brain imaging features derived from MRI, DTI, and rs‐fMRI yielded higher brain age prediction accuracy than single‐modal features. A new method to correct the bias of the brain age gap was proposed, which accounts for the nonlinearity and gender difference of the brain development trajectory. The brain age was found to share a large variance with behavioral scores. The corrected brain age gap was uncorrelated with chronological age and was highly associated with behavioral performance. The bias correction of brain age gap also removed potential confounding factors that may cause the altered brain development of the patients with disorders.

This study was a follow‐up to the initial work (Liang et al., 2019) to combine three modalities of *T*_1_‐weighted MRI, DTI and resting‐state fMRI features in brain age prediction. In ML, noninformative features often deteriorate prediction performance on an independent validation set. Thus, more features do not guarantee better prediction performance. Though the multimodal brain imaging features are likely to contain complementary information, combining all features derived from the three imaging modalities unpreventably introduces additional noise and increases the chance of overfitting. Thus, feature selection is a common practice to improve prediction performance, especially when a large number of features are included. Here we showed that combining multimodal brain imaging features improved prediction accuracy without additional feature selection procedures. By quantifying the prediction contribution of each feature in terms of the reduction of prediction accuracy when the feature was removed, we found age‐related brain changes are widely distributed across brain regions and are captured by both structural and functional imaging modalities. In addition, these features were consistent across different ML methods, especially for Ridge, SVR, and GPR. For example, most of the features contributed positively to brain age prediction were GMV, ALFF, and FA and most of the features contributed negatively to brain age prediction were the ReHo and MD. Interestingly, though the DTI features alone yielded worse prediction performance than rs‐fMRI features in single modality analysis, the DTI features yielded higher prediction accuracy than rs‐fMRI features when each type of features was combined with GMV features. This implies that the changes in the gray matter and white matter are likely to interact during brain development.

We also found a clear and consistent nonlinear trend of brain development across all analyses. These results support the idea that predicted brain age contained additional information on brain development and aging beyond chronological age. However, this nonlinear trend of brain age growth has been reported in only a few studies. One potential reason might be that subjects in most of the previous studies have a larger age range (Cole et al., 2015, 2017; Mwangi et al., 2013; Schnack et al., 2016). For a population with a larger range of age, the age‐related brain changes involve both maturation and aging effects which have opposite rate of changes. Specifically, the maturation process is fast in youth and gets slower for adults and elderly people, whereas the aging process is slow for youth and adults and gets faster for elderly people. When an ML model was built on subjects across a wider life span, the opposite trends of brain changes were rarely taken into consideration. In addition, though we propose *R* ^2^ of the nonlinear fitting was a better way to quantify the brain age prediction performance, it is worth noting that the improvement is relatively small, thus it does not change the ranking of the different ML models in terms of prediction accuracy. We chose the quadratic regression model to account for nonlinear brain development trajectory. The nonlinear trend shown in Figures 3, 4, and 5 also supported our modeling strategy. Because ages ranged from 8 to 21 for subjects in the PNC study, we did not expect a more complex brain development trajectory that may occur across a wider life span. Nonparametric methods hold great potential for modeling brain development trajectory due to their flexibility to approximate complex curves without assuming any specific parametric form. Compared to more complex modeling approach, the quadratic regression offered a good balance between model complexity and interpretability. Future research is needed to evaluate the application of nonparametric methods in brain age estimates.

Our results provided the first direct comparison between brain age and chronological age in terms of their relationship to behavioral performance. As true brain age is unknown and is likely to deviate from chronological age, regression based metrics (e.g., correlation coefficient, MAE, and *R* ^2^) are limited. Thus, additional validation measures such as cognitive behavioral scores are essential for comparing different brain age prediction models. We showed that brain age was significantly related to behavioral performance. However, we failed to find a higher variance explained by behavioral performance for brain age than chronological age. One potential reason might be that the high level of noise in brain imaging data could be detrimental to the brain age prediction accuracy. Besides, some behavioral scores in the CNB might not be related to brain development but postnatal experiences, which are more closely related to chronological age. We also examined whether the bias‐corrected brain age gap was related to CNB scores. Our result showed that with the brain age controlled, face memory (FMEM) accuracy and sensory‐motor (SM) speed scores were positively related to the brain age gap. This is consistent with a previous study (Erus et al., 2015). Additionally, we found the language (LAN) accuracy scores, attention (ATT), and verbal memory (VMEM) speed scores were negatively related to the brain age gap. Interestingly, we found some behavioral scores exhibited the opposite direction in their relationship with the brain age gap and brain age/chronological age. For example, SM speed score was negatively related to the brain age and chronological age but positively related to the brain age gap, whereas the LAN accuracy score was positively related to the brain age gap and chronological age but negatively related to brain age gap. Considering the bias‐corrected brain age gap is orthogonal to chronological age, the opposite relationships are unlikely due to the systematic bias (i.e., negative correlation between brain age gap and chronological age). One potential explanation is that brain age gap contains information on both brain development and aging, even for youth. The decomposition of the two opposite components in brain age gap is of great challenge and importance in the future work.

We also showed that bias correction removed the confounding effects of chronological age and gender when brain age gap was compared between the HC and disorder groups. For example, the uncorrected brain age gap of the specific phobia group was significantly higher than that of the HC. However, there was no significant difference in bias‐corrected brain age gap between the two groups. In the comparison of the HC and PTSD groups, there was a nonsignificant difference in the brain age gap between the two groups without bias correction. But it was significant with bias correction (Formula (1)). Since the PTSD group had more female subjects compared to the HC group, it is possible that the unbalanced distribution of age and gender in the PTSD group could introduce potential confounding effects. This is partially supported by a marginally significant bias‐corrected brain age gap including the interaction between chronological age and gender (Formula (2)).

Although the PTSD group showed higher brain age gap, it was not related to better behavioral performance compared to the HC group. Results from stepwise regression analysis showed that brain development of the HCs and anxiety disorder groups were differentially related to the behavioral changes. Based on the coefficients estimated in the stepwise regression models, we see the accuracy and speed scores of EDI task increased with the brain age for HCs, but not for specific phobia and PTSD groups. This is consistent with previous studies indicating emotional dysregulation on anxiety disorders (Etkin & Wager, 2007). Furthermore, we also showed that with the brain age controlled, a higher brain age gap was associated with better performance in EDI for the HCs but not for the anxiety disorder groups. The anxiety disorder groups showed deteriorated performance on social cognition and complex cognition tasks with increasing brain age. In summary, our results suggest a slower or even reversed cognitive development in the anxiety disorder groups, and that their brain age may reveal more about aging than development.

Compared to traditional ML methods, one advantage of using DNN in brain age prediction is to build the model directly with the raw imaging data (Cole et al., 2017; LeCun et al., 2015). In addition, DNN is able to learn more complex nonlinear brain growth patterns with hierarchical structure progressively coding representations from simple to complex (Hazlett et al., 2017). Our result showed similar performance in brain age prediction between DNN and other traditional ML methods. With an increasing number of samples and more powerful computing resources for more layers, DNN may show superiority over the traditional ML methods. However, the low interpretability of DNN limits its application.

In conclusion, our result suggests brain‐based age prediction benefits from multimodal imaging features. Brain age shows a nonlinear development trajectory with gender difference. It is also closely associated with behavioral performance. This association for the anxiety disorder patients is altered compared to the HCs, which may reflect accelerated aging of their brain. The brain age of the PTSD patients is older than that of the HCs and it is related to deficits in the performance of social cognition tasks. As the age range of our research is limited, future studies can examine the effectiveness of bias correction on a wider age range and explore how to build ML models to dissociate the maturity and aging effects of the brain. Additionally, to what extent genetics determines the brain development trajectory is also an intriguing topic.

## Supporting information


**Appendix** S1: Supporting Information

## Data Availability

Data used in this study are publicly available through the database of Genotypes and Phenotypes (dbGaP). The R and Python codes for implementing machine learning methods in the paper are available upon request.
