# Supplementary material for: Improved prediction of brain age using multimodal neuroimaging data
Source: Hum Brain Mapp. 2019 Dec 14;41(6):1626–43. doi: 10.1002/hbm.24899 (PMC7267976; doi:10.1002/hbm.24899)
Supplement: Supplementary file 1 — Appendix S1: Supporting Information [file HBM-41-1626-s001.pdf]

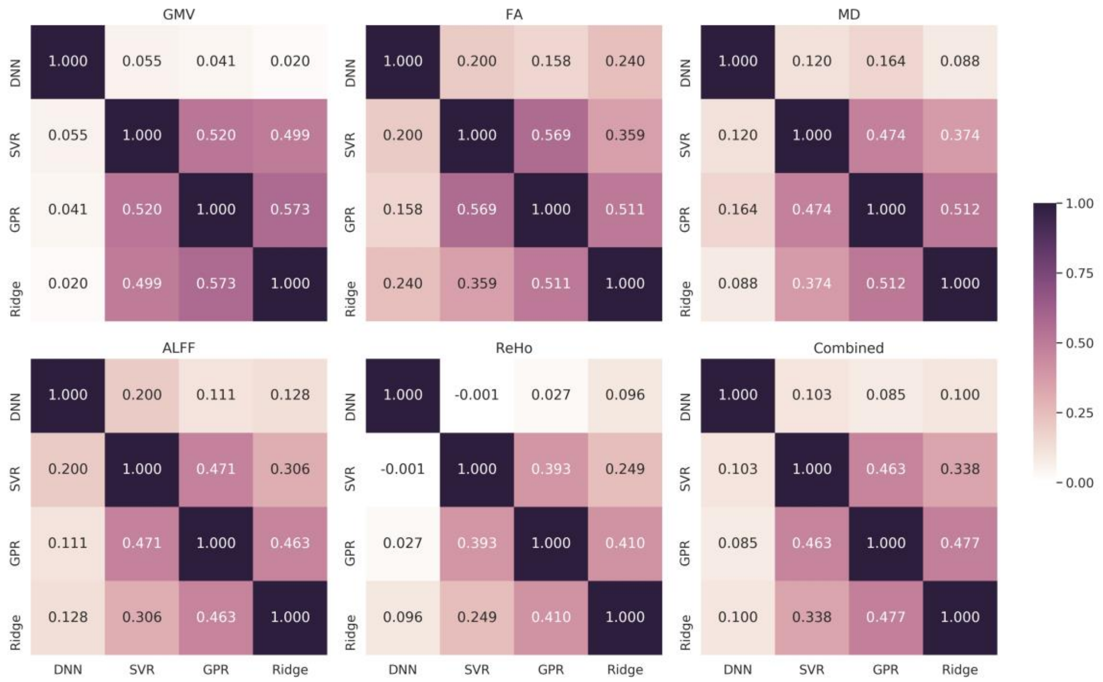

Figure S1. Correlation of feature importance across different ML methods. The heatmaps depict the correlation coefficient (Kendall's Tau) of feature importance across brain regions for each pair of the four ML methods. For DNN, SVR and GPR, feature importance was computed as the reduction of prediction accuracy by removing one feature at a time. For ridge regression, feature importance was calculated as the absolute feature weight.

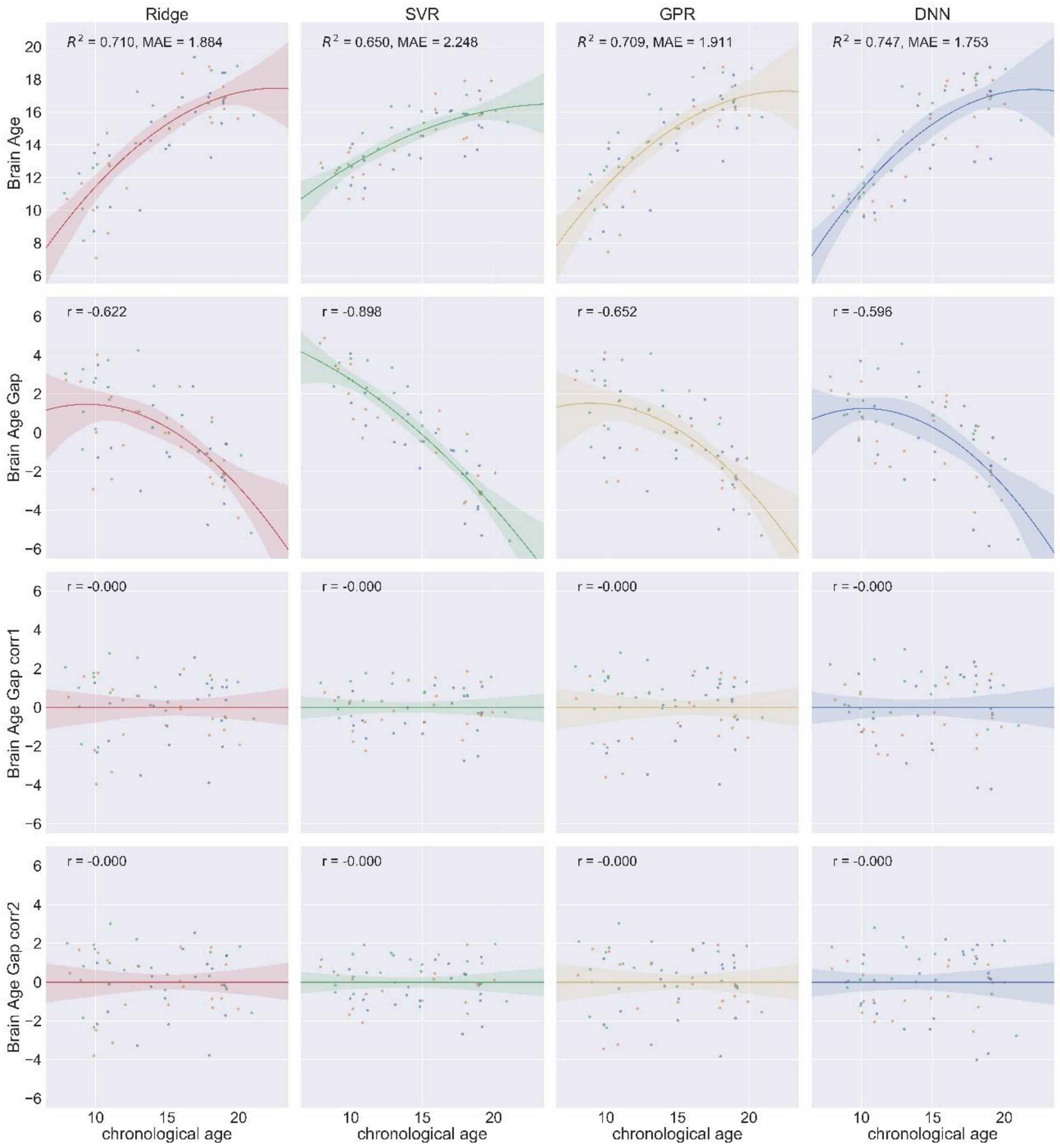

Figure S2. Scatterplots of brain age/brain age gap (y-axis) and chronological age (x-axis) for each of the four ML models. The first row shows a positive correlation between chronological age and brain age estimated from multimodal brain imaging features. Color indicates samples in different folds of cross-validation. The second row shows a negative correlation between the brain age gap (predicted brain age – chronological age) and chronological age. The third and fourth rows show the corrected brain age gap is orthogonal to chronological age. The corrected brain age gap is the difference between the brain age and the fitted brain age with a quadratic function of chronological age, gender, and with (fourth row) or without (third row) their interaction terms.

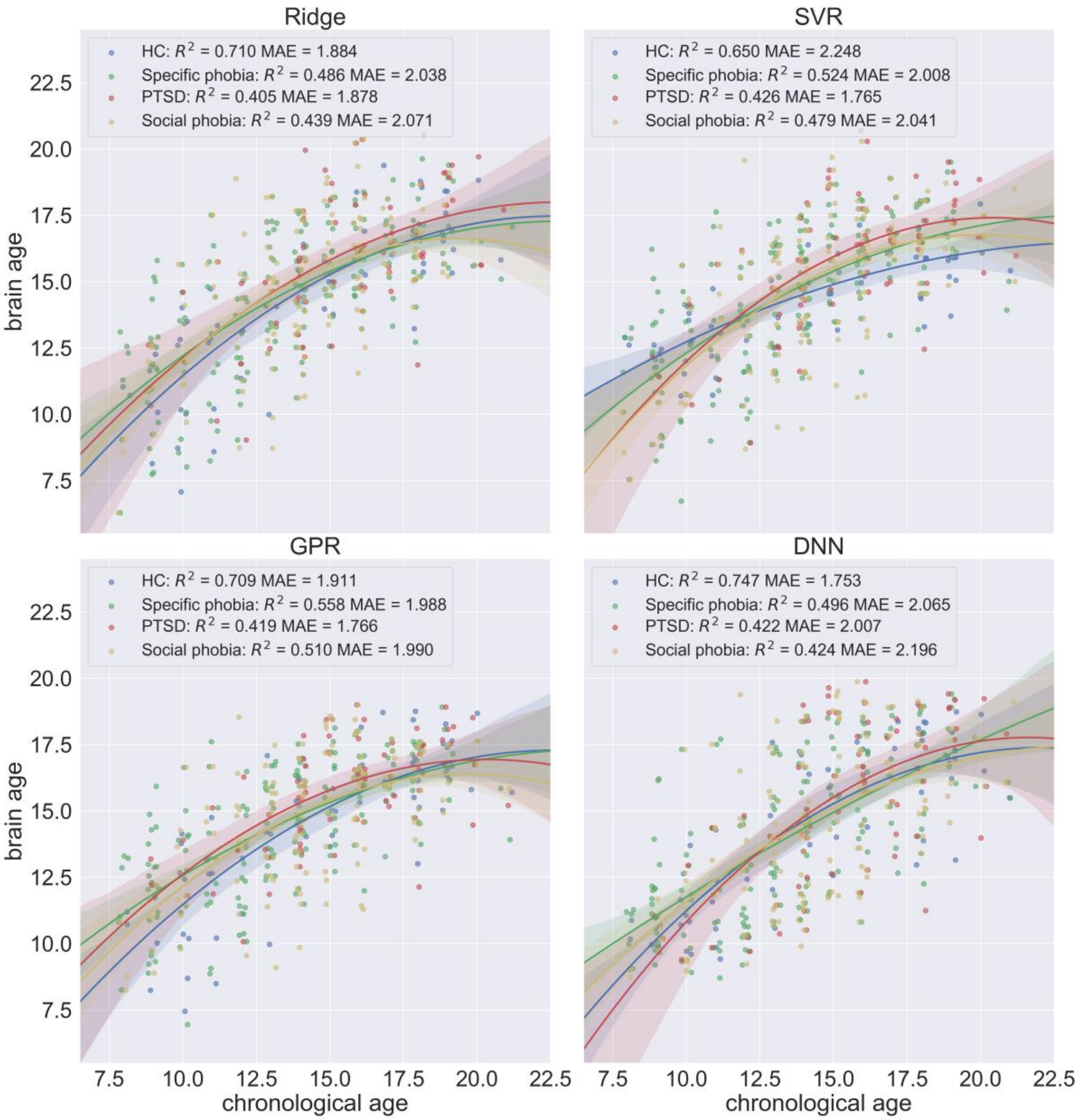

Figure S3. Scatterplots of chronological age and brain age estimated by (a) ridge regression, (b) support vector regression, (c) Gaussian process regression, and (d) deep neural networks for the HC and anxiety disorder groups. The shaded area along the regression curve indicates the 95% bootstrapped confidence interval.

Table S1. GMV features ranked among the top 100 predictive neuroimaging features for brain age prediction across the four machine learning models.

ROI	DNN	SVR	GPR	Ridge
Right Precuneus	✓	✓	✓	✓
Right Hippocampus	✓	✓	✓	✓
Right Pallidum	✓	✓	✓	✓
Left Precuneus	✓	✓	✓	✓
Left Hippocampus	✓	✓	✓	✓
Left Pallidum	✓	✓	✓	✓
Right Posterior Cingulate Gyrus	✓	✓	✓	✓
Right Temporal Pole	✓	✓	✓	✓
Right Posterior Insula	✓	✓	✓	✓
Right Ventral Ventricle		✓	✓	✓
Right Cerebellar Lobule Cerebellar Vermal Lobules VI-VII	✓		✓	✓
Right Angular Gyrus	✓		✓	✓
Left Ventral Ventricle		✓	✓	✓
Left Inferior Frontal Angular Gyrus	✓		✓	✓
Left Third Ventricle	✓	✓		✓
Left Frontal Operculum	✓	✓	✓	
Right Optic Chiasm	✓		✓	✓
Left Cerebellar Lobule Cerebellar Vermal Lobules I-V	✓		✓	✓
Left Middle Frontal Gyrus	✓		✓	✓
Right Parahippocampus Gyrus	✓	✓		✓
Right Brainstem		✓	✓	✓
Left Supramarginal Gyrus		✓	✓	✓
Right Occipital Pole	✓		✓	✓
Left Putamen		✓	✓	✓
Right Putamen		✓	✓	✓
Right Anterior Cingulate Gyrus	✓		✓	
Right Inferior Frontal Angular Gyrus		✓	✓	
Right Supramarginal Gyrus		✓		✓
Right Gyrus Rectus	✓			✓
Right Postcentral Gyrus	✓			✓
Left Brainstem		✓	✓	
Left Inferior Frontal Gyrus	✓	✓		
Left Fusiform Gyrus	✓		✓	
Left Medial Frontal Cerebrum	✓		✓	
Right Amygdala			✓	✓
Left Fourth Ventricle		✓		
Left Parahippocampus Gyrus	✓			
Left Gyrus Rectus	✓			

Left Frontal Pole	✓			
Left Planum Polare			✓	
Left Posterior Insula	✓			
Right Frontal Pole	✓			
Left Superior Temporal Gyrus		✓		
Left Temporal Transverse Gyrus	✓			
Right Lateral Orbital Gyrus	✓			
Right Lingual Gyrus	✓			
Left Medial Postcentral Gyrus	✓			
Right Inferior Lateral Ventricle		✓		
Right Parietal Operculum	✓			
Right Inferior Occipital Gyrus	✓			
Left Lateral Orbital Gyrus	✓			
Right Middle Cingulate Gyrus	✓			
Right Cerebellar Lobule Cerebellar Vermal Lobules VIII-X	✓			
Right Third Ventricle		✓		
Left Postcentral Gyrus	✓			
Right Inferior Frontal Gyrus	✓			
Right Fusiform Gyrus	✓			
Right Anterior Orbital Gyrus	✓			
Left Basal Cerebrum and Forebrain Brain	✓			
Left Anterior Cingulate Gyrus	✓			
Left Occipital Fusiform Gyrus	✓			
Left Entorhinal Area	✓			
Right Anterior Insula	✓			
Right Calcarine and Cerebrum	✓			
Right Superior Medial Frontal Gyrus	✓			
Right Inferior Frontal Orbital Gyrus	✓			
Left Inferior Occipital Gyrus	✓			
Right Basal Cerebrum and Forebrain Brain	✓			
Left Angular Gyrus	✓			
Right Medial Precentral Gyrus	✓			
Left Cuneus	✓			
Right Entorhinal Area	✓			
Right Cuneus	✓			
Right Central Operculum	✓			
Right Medial Postcentral Gyrus	✓			
Left Superior Medial Frontal Gyrus	✓			
Left Anterior Orbital Gyrus	✓			
Left Middle Temporal Gyrus	✓			
Left Middle Cingulate Gyrus	✓			

Left Middle Occipital Gyrus	✓			
Right Medial Frontal Cerebrum	✓			
Left Central Operculum	✓			
Left Parietal Operculum	✓			
Left Occipital Pole	✓			
Right Temporal Transverse Gyrus	✓			
Right Occipital Fusiform Gyrus	✓			
Left Medial Precentral Gyrus	✓			
Right Middle Temporal Gyrus	✓			
Right Middle Frontal Gyrus	✓			
Left Anterior Insula	✓			
Left Calcarine and Cerebrum	✓			
Left Posterior Cingulate Gyrus	✓			
Left Inferior Frontal Orbital Gyrus	✓			
Right Superior Frontal Gyrus				✓

Note. ✓ in a cell indicates that a GMV feature is ranked among the top 100 predictive neuroimaging features by a machine learning model.

Table S2. FA features ranked among the top 100 predictive neuroimaging features for brain age prediction across the four machine learning models.

ROI	DNN	SVR	GPR	Ridge
Body of corpus callosum	✓	✓	✓	✓
Left Corticospinal tract		✓	✓	✓
Right External capsule		✓	✓	✓
Left Inferior longitudinal fasciculus		✓	✓	✓
Right Anterior limb of internal capsule		✓	✓	✓
Left Inferior cerebellar peduncle		✓	✓	✓
Left Corticospinal tract		✓	✓	✓
Left Posterior thalamic radiation (include optic radiation)		✓	✓	✓
Right Corticospinal tract		✓	✓	✓
Right Superior cerebellar peduncle		✓		✓
Left Posterior corona radiata			✓	✓
Right Medial lemniscus			✓	✓
Middle cerebellar peduncle	✓			
Right Superior longitudinal fasciculus		✓		
Genu of corpus callosum	✓			
Left Anterior corona radiata			✓	
Left External capsule				✓

Note. ✓ in a cell indicates that an FA feature is ranked among the top 100 predictive neuroimaging features by a machine learning model.

Table S3. MD features ranked among the top 100 predictive neuroimaging features for brain age prediction across the four machine learning models.

ROI	DNN	SVR	GPR	Ridge
Left Cerebral peduncle		✓	✓	✓
Left Posterior limb of internal capsule		✓	✓	✓
Left Posterior corona radiata		✓	✓	✓
Left Cingulum (cingulate gyrus)		✓	✓	✓
Right Cerebral peduncle		✓	✓	✓
Right Posterior thalamic radiation (include optic radiation)		✓	✓	✓
Right Inferior fronto-occipital fasciculus		✓		✓
Left Inferior fronto-occipital fasciculus		✓		✓
Forceps minor			✓	✓
Fornix (column and body of fornix)			✓	✓
Right Posterior corona radiata			✓	✓
Left Superior fronto-occipital fasciculus		✓		
Left External capsule		✓		
Right Cingulum (cingulate gyrus)				✓
Left Anterior corona radiata				✓
Left Corticospinal tract				✓

Note. ✓ in a cell indicates that an MD feature is ranked among the top 100 predictive neuroimaging features by a machine learning model.

Table S4. ALFF features ranked among the top 100 predictive neuroimaging features for brain age prediction across the four machine learning models.

ROI	DNN	SVR	GPR	Ridge
Right Amygdala, medial amygdala	√	√	√	√
Right Inferior frontal gyrus, caudal area 45	√	√	√	√
Right Cingulate gyrus, pregenual area 32		√	√	√
Left Insular gyrus, dorsal agranular insula	√	√		√
Left Inferior frontal gyrus, dorsal area 44		√	√	√
Left Fusiform gyrus, lateroventral area 37		√	√	√
Left Inferior parietal lobule, caudal area 40 (PFm)		√	√	√
Left Superior frontal gyrus, medial area 9		√	√	√
Left Superior temporal gyrus, medial area 38		√	√	√
Right Basal ganglia, nucleus accumbens		√	√	√
Left Orbital gyrus, lateral area 12/47		√	√	√
Right Inferior temporal gyrus, extreme lateroventral area 37		√	√	√
Left Superior parietal lobule, lateral area 5		√	√	√
Left Inferior frontal gyrus, rostral area 45		√	√	√
Left Insular gyrus, dorsal dysgranular insula	√	√		
Right Thalamus, occipital thalamus	√			√
Right Orbital gyrus, orbital area 12/47			√	√
Left Inferior frontal gyrus, ventral area 44			√	√
Right Orbital gyrus, lateral area 11			√	√
Left Orbital gyrus, orbital area 12/47		√		√
Right Inferior frontal gyrus, rostral area 45		√	√	
Left Inferior frontal gyrus, opercular area 44			√	√
Right Thalamus, caudal temporal thalamus		√		
Right Parahippocampal gyrus, area TL (lateral PPHC		√		
Right Superior frontal gyrus, medial area 9		√		
Left Thalamus, medial pre-frontal thalamus		√		
Left Superior frontal gyrus, lateral area 9		√		
Right Fusiform gyrus, medioventral area 37			√	
Right Cingulate gyrus, rostroventral area 24			√	
Right Superior frontal gyrus, lateral area 9			√	
Left Thalamus, pre-motor thalamus				√
Right Postcentral gyrus, area 1/2/3(tongue and larynx region)				√
Left Insular gyrus, dorsal granular insula				√
Left Superior frontal gyrus, medial area 10		√		

Left Insular gyrus, ventral agranular insula		✓		
Left Paracentral lobule, area (lower limb region)		✓		
Right Thalamus, medial pre-frontal thalamus		✓		
Left Thalamus, occipital thalamus	✓			
Left Insular gyrus, ventral dysgranular and granular insula	✓			
Right Insular gyrus, dorsal dysgranular insula	✓			
Left Thalamus, rostral temporal thalamus	✓			
Right Insular gyrus, dorsal agranular insula	✓			
Right Thalamus, rostral temporal thalamus	✓			
Left Thalamus, posterior parietal thalamus	✓			
Right Hippocampus, rostral hippocampus	✓			
Right Thalamus, posterior parietal thalamus	✓			
Right Insular gyrus, ventral dysgranular and granular insula	✓			
Right Insular gyrus, hypergranular insula	✓			
Left Insular gyrus, hypergranular insula	✓			
Left Thalamus, caudal temporal thalamus	✓			
Right Basal ganglia, ventral caudate		✓		
Left Amygdala, medial amygdala		✓		
Left Inferior temporal gyrus, intermediate lateral area 20				✓

Note. ✓ in a cell indicates that an ALFF feature is ranked among the top 100 predictive neuroimaging features by a machine learning model. Details of the BN 246 atlas label information can be found at the link below.

https://atlas.brainnetome.org/bnatlas.html

Table S5. ReHo features ranked among the top 100 predictive neuroimaging features for brain age prediction across the four machine learning models.

ROI	DNN	SVR	GPR	Ridge
Left Superior temporal gyrus, rostral area 22	✓	✓	✓	✓
Left Superior frontal gyrus, medial area 10		✓	✓	✓
Right Precentral gyrus, caudal dorsolateral area 6		✓	✓	✓
Right Hippocampus, rostral hippocampus		✓	✓	✓
Left Postcentral gyrus, area 1/2/3(trunk region)		✓	✓	✓
Left Middle frontal gyrus, lateral area 10		✓	✓	✓
Left Basal ganglia, ventromedial putamen		✓	✓	✓
Right Insular gyrus, dorsal agranular insula		✓	✓	✓
Right Superior temporal gyrus, A41/4 area 41/42		✓	✓	✓
Right Parahippocampal gyrus, caudal area 35/36		✓	✓	✓
Right Superior parietal lobule, lateral area 5		✓	✓	✓
Left Superior frontal gyrus, medial area 8			✓	✓
Left Cingulate gyrus, pregenual area 32			✓	✓
Right Precuneus, dorsomedial parietooccipital sulcus(PEr)		✓	✓	
Right Superior temporal gyrus, rostral area 22		✓	✓	
Left Precentral gyrus, caudal ventrolateral area 6			✓	✓
Right Cingulate gyrus, dorsal area 23			✓	✓
Right Inferior parietal lobule, rostroventral area 40(PFop)			✓	✓
Right Postcentral gyrus, area 1/2/3(trunk region)		✓	✓	
Left Middle frontal gyrus, ventrolateral area 8			✓	✓
Left Orbital gyrus, orbital area 12/47		✓	✓	
Right Cingulate gyrus, subgenual area 32			✓	✓
Left Orbital gyrus, A1 area 13			✓	
Left Inferior temporal gyrus, rostral area 20			✓	
Left Middle temporal gyrus, rostral area 21				✓
Right Middle frontal gyrus, inferior frontal junction			✓	
Left Superior parietal lobule, postcentral area 7			✓	
Left Thalamus, lateral pre-frontal thalamus			✓	
Right Cingulate gyrus, rostroventral area 24				✓
Left Cingulate gyrus, ventral area 23		✓		
Right Precentral gyrus, area 4(tongue and larynx region)			✓	
Right Inferior parietal lobule, rostrodorsal area 40(PFt)		✓		
Right Middle frontal gyrus, dorsal area 9/46		✓		

Left Inferior frontal gyrus, dorsal area 44		✓		
Right Lateral occipital cortex, medial superior occipital gyrus		✓		
Left Superior parietal lobule, caudal area 7		✓		
Right Lateral occipital cortex, lateral superior occipital gyrus		✓		
Left Superior temporal gyrus, A41/4 area 41/42		✓		
Right Parahippocampal gyrus, entorhinal cortex)		✓		
Right Superior parietal lobule, intraparietal area 7(hIP3)		✓		
Left Precuneus, dorsomedial parietooccipital sulcus(PEr)		✓		
Left Middle frontal gyrus, A4 area 46				✓

Note. ✓ in a cell indicates that a ReHo feature is ranked among the top 100 predictive neuroimaging features by a machine learning model. Details of the BN 246 atlas label information can be found at the link below.

https://atlas.brainnetome.org/bnatlas.html
